# Synergistic interactions between anammox and dissimilatory nitrate reducing bacteria sustains reactor performance across variable nitrogen loading ratios

**DOI:** 10.3389/fmicb.2023.1243410

**Published:** 2023-08-09

**Authors:** Christian White, Edmund Antell, Sarah L. Schwartz, Jennifer E. Lawrence, Ray Keren, Lijie Zhou, Ke Yu, Weiqin Zhuang, Lisa Alvarez-Cohen

**Affiliations:** ^1^Department of Civil & Environmental Engineering, University of California, Berkeley, Berkeley, CA, United States; ^2^CDM Smith, Boston, MA, United States; ^3^College of Chemistry and Environmental Engineering, Shenzhen University, Shenzhen, China; ^4^School of Environment and Energy, Shenzhen Graduate School, Peking University, Shenzhen, China; ^5^Department of Civil & Environmental Engineering, University of Auckland, Auckland, New Zealand; ^6^Earth and Environmental Sciences Division, Lawrence Berkeley National Laboratory, Berkeley, CA, United States

**Keywords:** anammox, DNRA, denitrification, metagenome, stoichiometric ratio, bioreactor, wastewater

## Abstract

Anaerobic ammonium oxidizing (anammox) bacteria are utilized for high efficiency nitrogen removal from nitrogen-laden sidestreams in wastewater treatment plants. The anammox bacteria form a variety of competitive and mutualistic interactions with heterotrophic bacteria that often employ denitrification or dissimilatory nitrate reduction to ammonium (DNRA) for energy generation. These interactions can be heavily influenced by the influent ratio of ammonium to nitrite, NH_4_^+^:NO_2_^−^, where deviations from the widely acknowledged stoichiometric ratio (1:1.32) have been demonstrated to have deleterious effects on anammox efficiency. Thus, it is important to understand how variable NH_4_^+^:NO_2_^−^ ratios impact the microbial ecology of anammox reactors. We observed the response of the microbial community in a lab scale anammox membrane bioreactor (MBR) to changes in the influent NH_4_^+^:NO_2_^−^ ratio using both 16S rRNA gene and shotgun metagenomic sequencing. Ammonium removal efficiency decreased from 99.77 ± 0.04% when the ratio was 1:1.32 (prior to day 89) to 90.85 ± 0.29% when the ratio was decreased to 1:1.1 (day 89–202) and 90.14 ± 0.09% when the ratio was changed to 1:1.13 (day 169–200). Over this same timespan, the overall nitrogen removal efficiency (NRE) remained relatively unchanged (85.26 ± 0.01% from day 0–89, compared to 85.49 ± 0.01% from day 89–169, and 83.04 ± 0.01% from day 169–200). When the ratio was slightly increased to 1:1.17–1:1.2 (day 202–253), the ammonium removal efficiency increased to 97.28 ± 0.45% and the NRE increased to 88.21 ± 0.01%. Analysis of 16 S rRNA gene sequences demonstrated increased relative abundance of taxa belonging to Bacteroidetes, Chloroflexi, and Ignavibacteriae over the course of the experiment. The relative abundance of Planctomycetes, the phylum to which anammox bacteria belong, decreased from 77.19% at the beginning of the experiment to 12.24% by the end of the experiment. Analysis of metagenome assembled genomes (MAGs) indicated increased abundance of bacteria with *nrfAH* genes used for DNRA after the introduction of lower influent NH_4_^+^:NO_2_^−^ ratios. The high relative abundance of DNRA bacteria coinciding with sustained bioreactor performance indicates a mutualistic relationship between the anammox and DNRA bacteria. Understanding these interactions could support more robust bioreactor operation at variable nitrogen loading ratios.

## 1. Introduction

Nitrogen removal from wastewater is paramount to the remediation of anthropogenic nutrient pollution and the protection of sensitive aquatic environments (USEPA, 2007; Smith and Schindler, 2009; Davidson et al., 2014). One strategy to remove nitrogen from wastewater is anaerobic ammonium oxidation (anammox), in which ammonium (NH_4_^+^) is anaerobically oxidized using nitrite (NO_2_^−^) as an electron acceptor (Mulder et al., 1995; van de Graaf et al., 1995; Strous et al., 1998). Anammox is a biogeochemical process facilitated by chemolithoautotrophic bacteria in the Phylum Planctomycetes (Strous et al., 1999; Schmid et al., 2000) and is estimated to account for up to 70% of fixed nitrogen removal in marine environments (Kuypers et al., 2003; Devol, 2015). The anammox process has shown great potential for robust sidestream nitrogen removal, with full scale installations demonstrating nitrogen removal efficiencies up to 90% and ammonium removal rates up to 9.5 kg NH_4_^+^-N/L·d (Abma et al., 2007; Joss et al., 2009; Lackner et al., 2014) while using 60% less energy and producing 90% less sludge than conventional nitrification–denitrification systems (Jetten et al., 1997; Lackner et al., 2014; Cho et al., 2019). Despite these benefits, anammox-based water treatment faces multiple challenges including long start up periods (6 months-2 years) (Jetten et al., 2001; Kuenen, 2008; Kartal et al., 2013) and process instability due to inhibitory compounds (Jin et al., 2012; Lotti et al., 2012), operational fluctuations, and microbially-provoked destabilizations (Ali and Okabe, 2015). Thus, prior anammox research has sought to better-understand microbial community dynamics in order to design, maintain, and operate more resilient reactors.

Anammox reactors harbor phylogenetically and functionally diverse bacteria that engage in a variety of synergistic, competitive, and mutualistic interactions (Guo et al., 2016; Bhattacharjee et al., 2017; Lawson et al., 2017; Pereira et al., 2017). The presence of a core microbial community identified alongside anammox bacteria—which have never been isolated in pure culture—suggests an ecological niche specific to the conditions found inside of the reactor. Previous research consistently identifies bacteria belonging to the phyla Proteobacteria, Chloroflexi, Ignavibacteria, and Bacteroidetes as part of this core community (Gonzalez-Martinez et al., 2015; Keren et al., 2020). Many of the interactions occurring among different anammox community members are predicated on an exchange of organic carbon substrates, secondary metabolites, and various nitrogen species (Jenni et al., 2014; Zhao et al., 2018; Cao et al., 2020; Zhang et al., 2020, 2021). These complex relationships are key to improving nitrogen removal efficiency and sustaining microbial growth within bioreactors. For example, heterotrophic bacteria performing nitrate reduction through *nar* or *nap* nitrate reductases can reduce nitrate (NO_3_^−^) to nitrite, providing a substrate for anammox; anammox bacteria then produce organic carbon that feeds heterotrophic partners, forming a nitrite loop. However, other nitrogen metabolisms can disrupt this loop. Bacteria performing dissimilatory nitrate reduction to ammonium (DNRA) with *nrfA* or *nrfH* cytochrome nitrite reductases convert nitrite back to ammonium, forming antagonistic relationships with anammox bacteria that can disrupt reactor performance (Einsle et al., 2002; Speth et al., 2016; Wang et al., 2019). Heterotrophs performing DNRA actively compete with denitrifiers for organic carbon substrates and both nitrate and nitrite, a competition that has been shown to depend on C/N ratio, carbon source availability, and hydraulic retention time (van den Berg et al., 2016, 2017a,b).

The competition between bacteria performing DNRA and denitrification can also be altered by influent resource concentrations (Jia et al., 2020). In full scale systems, anammox is coupled with nitritation to provide a nitrite source, generally resulting in a nitrogen influent stream of approximately 50% ammonium (NH_4_^+^) and 50% nitrite (NO_2_^−^), or a NH_4_^+^:NO_2_^−^ molar ratio of 1:1 (Joss et al., 2009; Lackner et al., 2014). Because of the competition between denitrifying bacteria and DNRA for nitrite, the NH_4_^+^:NO_2_^−^ ratio needed within an anammox reactor tends to be higher. Highly enriched anammox cultures remove ammonium and nitrite at a ratio between 1:1.2 and 1:1.32 (Zhu et al., 2017). Any divergence from this ratio can lead to poor reactor performance, or in extreme cases, reactor crashes. While changes to reactor performance during ratio changes has been well documented, the underlying changes to the microbial community that drive performance changes are poorly understood.

Given that full-scale anammox reactors are susceptible to performance destabilization due to variable nitrogen loading (Joss et al., 2011), it is important to evaluate the effects of fluctuating nitrogen species ratios on the complex network of metabolic interdependencies between anammox, DNRA, and denitrification bacteria. Here we constrain the microbial community response to perturbations induced by influent nitrogen loading ratio changes in a lab scale anammox reactor. We evaluate the effects of variable nitrogen loading ratios on reactor performance, anammox activity, and microbial community dynamics; we also assess the changes in microbial interactions as a result of changing influent conditions using 16S rRNA amplicon sequencing and shotgun metagenomic sequencing analysis. The results provide insight into the competitive and synergistic relationships between bacteria employing different nitrogen metabolisms, and how these complex relationships affect reactor performance, stability, and resiliency.

## 2. Methods

### 2.1. Bioreactor operation

A 1 L anaerobic membrane bioreactor (MBR) was operated for 1 year prior to the experiment. The reactor was enriched for anammox bacteria from anaerobic digester solids. The specific details of initial inoculation and operation can be found in (Keren et al., 2020). A polyvinylidene fluoride membrane with a pore size of 0.22 μm was mounted to the inside of the reactor and a gas mix (Argon:CO_2_ = 95:5; 50 mL/min) was continuously supplied to purge the system of oxygen and maintain circumneutral pH (6.9–7.2) (Supplementary Figure S2). Temperature was maintained at 37°C using a heating jacket (Eppendorf, Hauppauge, NY) and mixing was provided through an impeller at a rate of 200 rpm. A synthetic media containing ammonium, nitrite, bicarbonate, and trace nutrients prepared anaerobically under nitrogen was continuously fed to the reactor; the exact composition can be found in Supplementary Table S1. Influent and effluent samples were collected every other day to monitor concentrations of ammonium, nitrite, and nitrate using HACH test kits (HACH, Loveland, CO), as described in the manufacturer’s methods 10031, 10019, and 10020, respectively. Mixed liquor suspended solids (MLSS) and mixed liquor volatile suspended solids (MLVSS) were measured according to standard methods (USEPA, 2001).

The experiment took place over 252 days during which the hydraulic retention time (HRT) of the reactor was maintained at 12 h (via an effluent pump) and the solids retention time (SRT) was maintained at 50 days (via biomass wasting). For the first 88 days of the experiment, NH_4_^+^and NO_2_^−^were loaded at the conventional 1:1.32 ratio at concentrations of 500 mg-N/L and 660 mg-N/L, respectively. On day 89, the influent ammonium concentration was raised to 600 mg-N/L, decreasing the NH_4_^+^:NO_2_^−^ ratio to 1:1.1. On day 169, the influent nitrite concentration was raised to 680 mg-N/L, increasing the NH_4_^+^:NO_2_^−^ ratio to 1:1.13. From day 200 to 252, the total nitrogen loading was slowly increased while also increasing the NH_4_^+^:NO_2_ ratio from 1:1.13 to 1:1.2 as shown in Table 1. This range of NH_4_^+^:NO_2_^−^ ratios was selected for the experiment in order to maintain stable operation of the reactor, as lower ratios (<1:1) have been demonstrated to lead to the accumulation of free ammonia (FA) (Fernández et al., 2012) and higher ratios (>1:1.3) have been shown to lead to nitrite inhibition (Jin et al., 2013). The dominant anammox strain in our MBR (*Brocadia sinica*) has also been shown to have higher sensitivity to nitrite inhibition than other strains such as *Kuenenia* (Oshiki et al., 2011). Thus the influent nitrogen ratios tested in this experiment were selected to avoid these issues.

**Table 1:** Influent nitrogen loading and NH_4_^+^:NO_2_^−^ ratio data.

Day	Influent ammonium (mg-N/L)	Influent nitrite (mg-N/L)	NH_4_^+^:NO_2_^−^ ratio	Nitrogen loading rate (g N/L-d)
0	500	660	1.32	2.32
89	600	660	1.1	2.52
169	600	680	1.13	2.52
200	600	700	1.17	2.6
217	600	710	1.18	2.62
218	600	720	1.2	2.64
235	640	768	1.2	2.82
243	660	792	1.2	2.9
250	680	816	1.2	2.99

### 2.2. DNA extraction

Biomass samples were collected every 2–10 days via syringe through an extraction port, flash frozen in liquid nitrogen, and stored at −80°C until further use. Genomic DNA was extracted from the samples using the DNeasy PowerSoil Kit (Qiagen, Carlsbad, CA) as described in the manufacturer’s protocol. DNA quality was assessed using a NanoDrop Spectrophotometer (Thermo Scientific, Waltham, MA) and Bioanalyzer 2100 (Agilent Technologies, Santa Clara, CA). DNA was quantified using a Qubit fluorometer (Thermofisher Scientific, Walthan, MA), diluted to 10 ng/μl with nuclease free water (Thermo Scientific, Waltham, MA), and stored at −20°C until further use. Shotgun metagenomic sequencing samples were sent to the Joint Genome Institute (JGI) in Walnut Creek, CA. There, DNA was sequenced (150 bp paired-end) on an Illumina HiSeq 2500 1 T sequencer (Ilumina, San Diego, CA). 16S rRNA sequencing for samples collected from day 1 to 45 were sequenced at the Institute for Environmental Genomics at the University of Oklahoma and the remaining samples were sequenced at JGI on an Illumina MiSeq sequencer (Illumina, San Diego, CA).

### 2.3. 16S rRNA gene analysis

The microbial community composition was evaluated by 16S ribosomal RNA sequencing of 28 DNA samples collected throughout the experiment. The V4 region was amplified using primers 515F (5′-GTGCCAGCMGCCGCGG-3′) and 806R (3′-TAATCTWTGGVHCATCAG-5′), with barcodes attached to the reverse primer. Amplicons were pooled at equal molarity and purified with the QIAquick Gel Extraction Kit (QIAGEN Sciences, Germantown, MD). Paired-end sequencing (250 bp paired-end) was then performed on the Illumina MiSeq sequencer (Illumina, San Diego, CA). The full protocol is provided by Wu et al. (2015). Sequence processing and data analysis was conducted using MOTHUR v.1.39.5, following the MiSeq Standard Operating Procedure (SOP) (Schloss et al., 2009), and OTUs were assigned based on a 97% sequence similarity threshold.

### 2.4. Metagenomic sequencing, assembly, and binning

Three DNA samples were used for metagenomic sequencing, two from single timepoints on day 37 and 140 and one bulked from samples taken on days 232, 235, and 237. Resulting sequences from each time point were processed separately according to the procedure previously reported in Keren et al. (2020). KEGG Automated Annotation Service (KAAS) was used to annotate predicted gene sequences using Hidden Markov Models (HMMs). Single time point genome abundances were calculated using reads per kilobase per million (RPKM). The log ratio change for each genome was calculated based on the procedure previously reported in Keren et al. (2020). Briefly, three genomes with stable coverage across the three time points were selected as reference frame genomes. The coverage of each genome was then divided by the coverage of the three reference frame genomes. These ratios were then used to calculate the log ratio changes between samples yielding three values, one for each reference frame genome. This was done in order to account/adjust for differences in sequencing depth between samples, which can otherwise lead to biased results. Relative replication rates were calculated using iRep (Brown et al., 2016). Briefly, the replication rates of bacteria were estimated by calculating the coverage ratio between the origin of replication and the terminus of replication. In a population that is not actively replicating, the coverage at the ratio and terminus will be the same, and the ratio will be one. For populations that are actively replicating the coverage will be greater around the origin of replication because of replication forks that have not finished replicating. Thus, the higher this ratio the higher the proportion of the population that is actively replicating.

### 2.5. Statistical analysis

Principal component analysis (PCA) was applied to evaluate the correlation between taxa abundance for different pathways (anammox, denitrification, and DNRA) and reactor performance parameters. In order to assign pathways to specific taxa, 16S rRNA sequences obtained from metagenomes with genes encoding for each pathway were aligned to representative sequences from amplicon sequencing following the procedure described in Keren et al. (2020). For MAGs not containing 16S rRNA sequences but classified down to the species level, sequences were obtained from NCBI. PCA analysis was conducted using R^1^ with the “factoextra” package in RStudio.^2^

## 3. Results

### 3.1. Bioreactor performance

**Figure 1:**
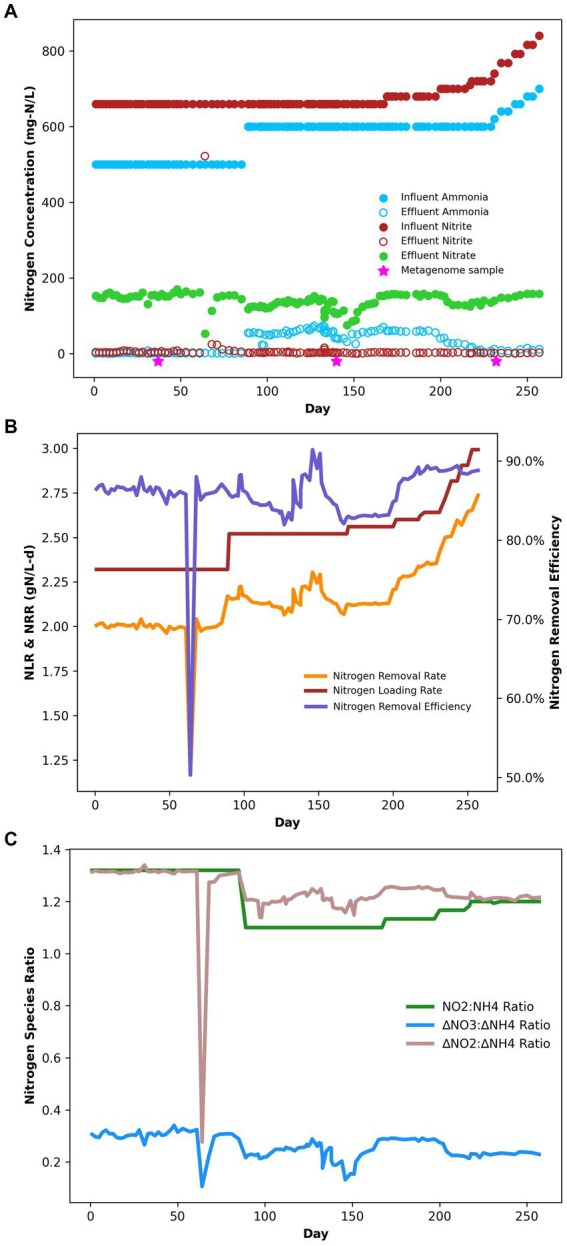
**(A)** Bioreactor performance nitrogen speciation data. **(B)** Bioreactor performance during the experiment in terms of NRR, NLR, and NRE. **(C)** Bioreactor performance stoichiometric ratios of nitrogen species.

Under the initial NH_4_^+^:NO_2_^−^ ratio of 1:1.32, the average ammonium and nitrite removal efficiencies were 99.77 ± 0.04% and 96.85 ± 2.31%, respectively (Table 2), and the average nitrogen removal rate (NRR) taking into consideration the generation of nitrate from nitrite by anammox bacteria to generate reducing equivalents for carbon fixation was 1.98 ± 0.03 g-N/L·d. This resulted in a nitrate production rate of 0.075 ± 0.002 g-N/L·d and a NO_3_^−^:NH_4_^+^ ratio of 0.298 ± 0.0007:1, which is slightly above the conventional ratio of 0.26:1 (Strous et al., 1998). Following the influent ratio shift on day 89, the average ammonium concentration in the effluent increased from 1.14 ± 0.22 mg-N/L to 55.19 ± 1.68 mg-N/L, which was expected due to the increased NH_4_^+^ in the influent. Of the 100 mg-N/L NH_4_^+^ added to the influent, about half was emitted in the effluent, while the remaining ammonium was likely removed through the anammox reaction or was assimilated for biomass synthesis. After increasing the NO_2_^−^ influent concentration, the nitrogen loading rate (NLR) was steadily increased from 2.60 g-N/L·d on day 200 to 2.99 g-N/L·d on day 250 (Table 1). From day 89 to day 202 the average ammonium removal efficiency fell to 90.74 ± 0.25% and the nitrite removal efficiency increased to 99.57 ± 0.04%; the NRR increased to 2.16 ± 0.01 g-N/L·d. This resulted in a nitrate production rate of 0.066 ± 0.002 g-N/L·d and a NO_3_^−^:NH_4_^+^ ratio of 0.24 ± 0.005:1. From day 202 to day 253, ammonium removal efficiency increased back to 97.28 ± 0.45%, and the NO_3_^−^:NH_4_^+^ ratio decreased again to 0.237 ± 0.005:1 (Figure 1).

**Table 2:** Membrane bioreactor (MBR) performance and effluent data.

Day	NH_4_^+^ Removal Efficiency	NO_2_^−^ Removal efficiency	Nitrogen removal efficiency	Nitrogen removal rate (g N/L-d)	Nitrate production rate (g N/L-d)	∆ NO_3_^–^:∆NH_4_^+^
0–89	99.77 ± 0.04%	96.85 ± 2.34%	85.26 ± 0.01%	1.98 ± 0.03	0.08 ± 0.01	0.30 ± 0.01
89–169	90.85 ± 0.29%	99.56 ± 0.05%	85.49 ± 0.01%	2.15 ± 0.01	0.06 ± 0.01	0.23 ± 0.01
169–200	90.14 ± 0.09%	99.65 ± 0.02%	83.04 ± 0.01%	2.13 ± 0.01	0.08 ± 0.01	0.29 ± 0.01
200–252	97.28 ± 0.45%	99.76 ± 0.03%	88.21 ± 0.01%	2.37 ± 0.03	0.07 ± 0.01	0.24 ± 0.05

### 3.2. Microbial community in MBR through 16S amplicon sequencing

**Figure 2:**
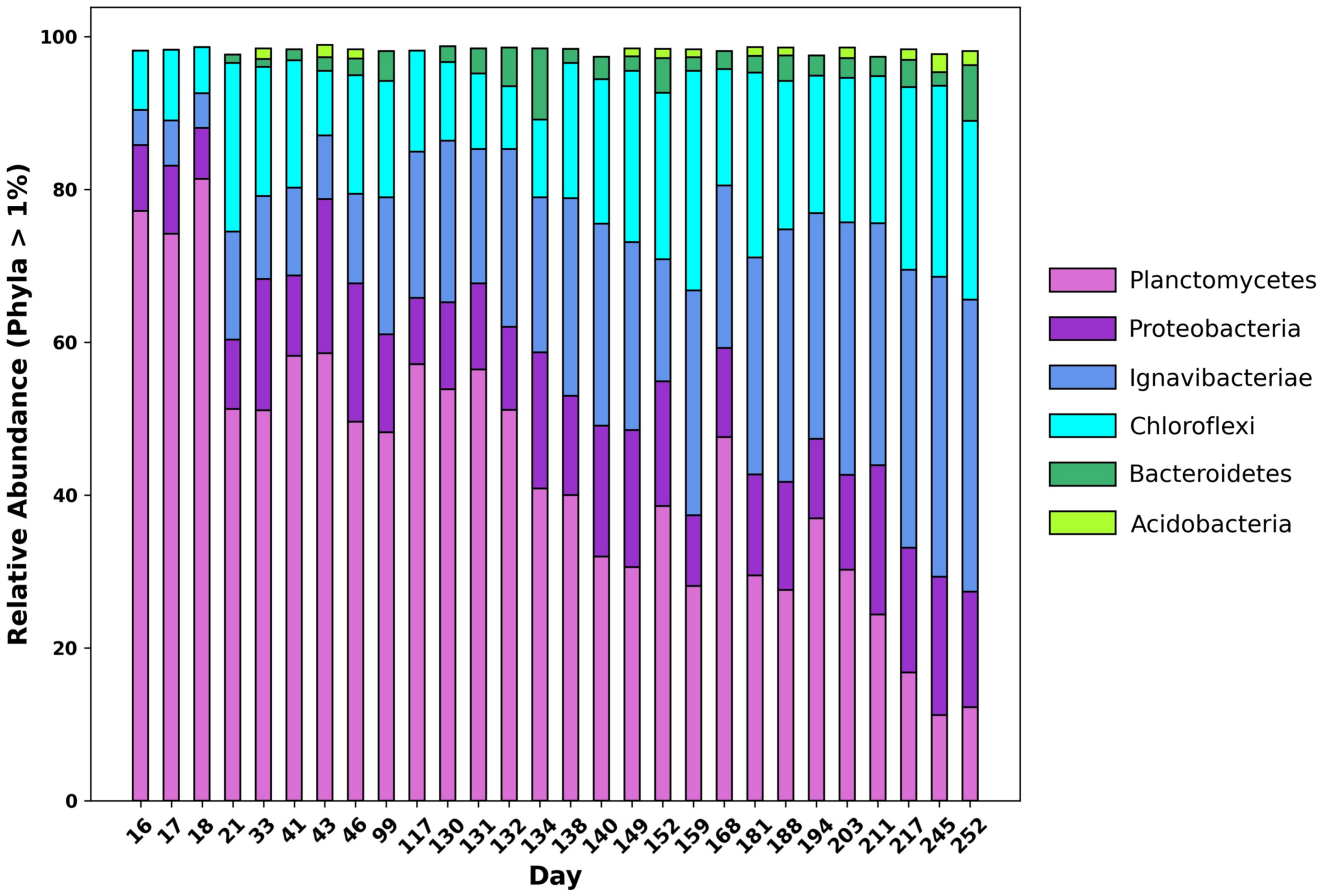
Changes in relative abundance at the phylum level of 16S rRNA amplicon sequences over time from day 16 of the experiment to day 252.

16S rRNA amplicon sequencing was conducted to ascertain changes in abundance of taxonomic groups throughout the reactor lifecycle. Figure 2 shows the relative abundance of 16S rRNA genes at the phylum level. At the beginning of the experiment, the dominant phylum in the reactor was Planctomycetes, accounting for 77.19% of total reads. Other significant phyla included Chloroflexi, Ignavibacteria, and Proteobacteria accounting for 7.73, 4.57, and 8.67% of total reads respectively, which is consistent with previously reported results (Pereira et al., 2017). Of the reads belonging to Chloroflexi, over 99% belonged to the class Anaerolineae and of the reads belonging to Proteobacteria, 50.40% belonged to the class Alphaproteobacteria. Over the course of the experiment, the relative abundance of Planctomycetes decreased from 77.19 to 12.24%. Meanwhile, the relative abundance of Chloroflexi and Ignavibacteria increased from 7.73 and 4.57% to 23.36 and 38.22%, respectively. The relative abundance of the phylum *Bacteroidetes* also increased from <0.05% at the beginning of the experiment to 7.30% by the end. The Shannon, Simpson, and Chao indices were calculated using relative abundance data from sequenced amplicons, which indicated that the microbial diversity of the community increased over the course of the experiment. Further information on diversity calculations and results can be found in the Supplementary Table S3.

**Figure 3:**
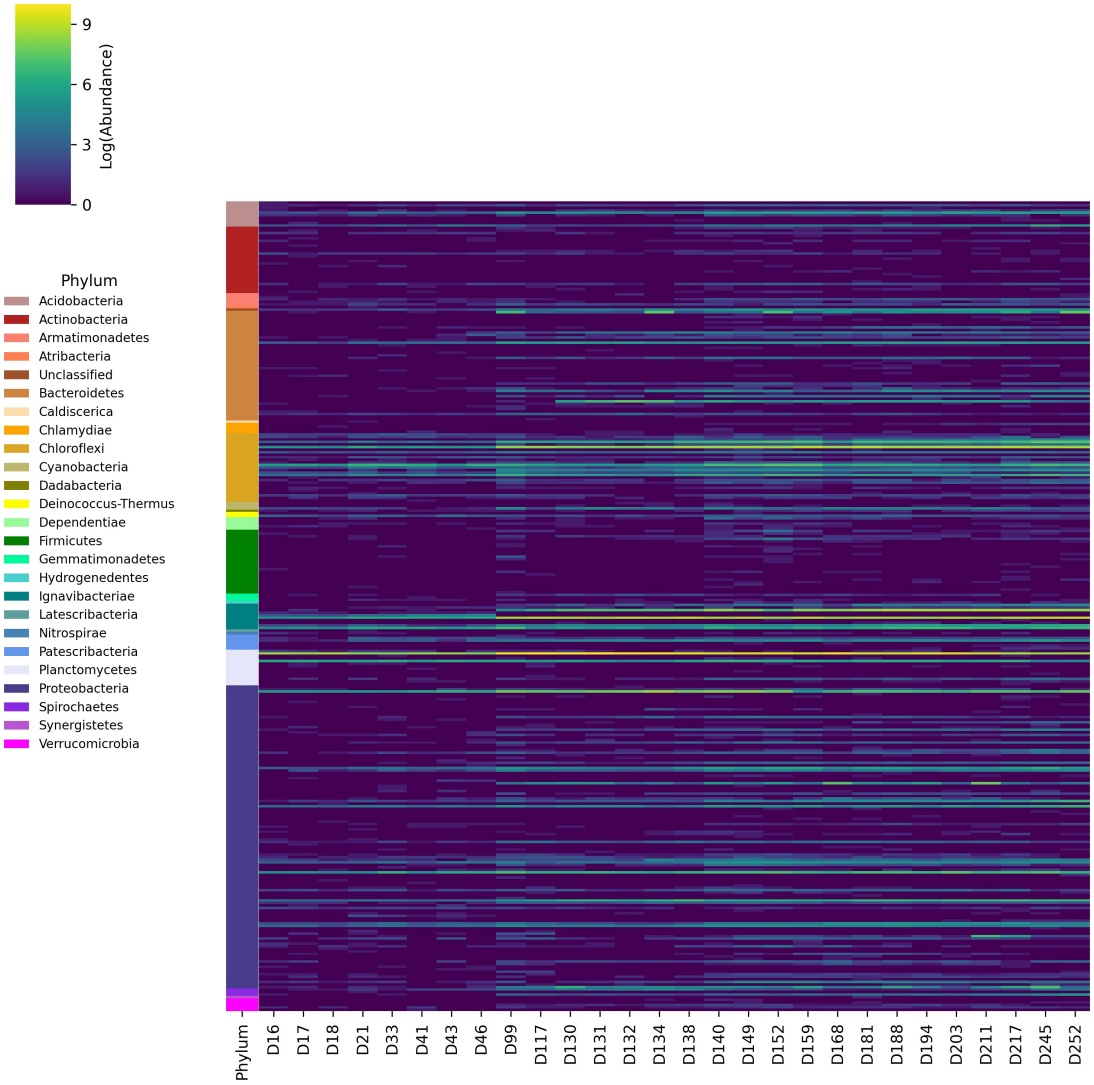
Changes in logarithmic abundances of 16S rRNA amplicon reads aggregated at the genus level over time from day 16 of the experiment to day 252.

The abundance of taxonomic groups was also aggregated at the genus level (Figure 3). These results are consistent with relative abundances at the phylum level, demonstrating high abundance genera in Chloroflexi, Ignavibacteria, Proteobacteria, and Bacteroidetes phyla. Several genera associated with the families Anaerolineaceae, Rhodocylacea, Burkholderiaceae and the order Ignavibacteriales were consistently abundant throughout the experiment, which was consistent with previously reported results (Du et al., 2017; Hu et al., 2018; Pereira et al., 2019; Xiao et al., 2021). Several of the genera from Anaerolineaceae and Ignavibacteriales increased by at least one order of magnitude following the initial ratio change made on day 89. Multiple genera from Rhodospiralles, Flavobacteriales, Sphingobacteriales, and Chitinophagales that were previously undetected or low abundance increased by at least two orders of magnitude post-ratio change.

### 3.3. Metagenome sequencing results

**Figure 4:**
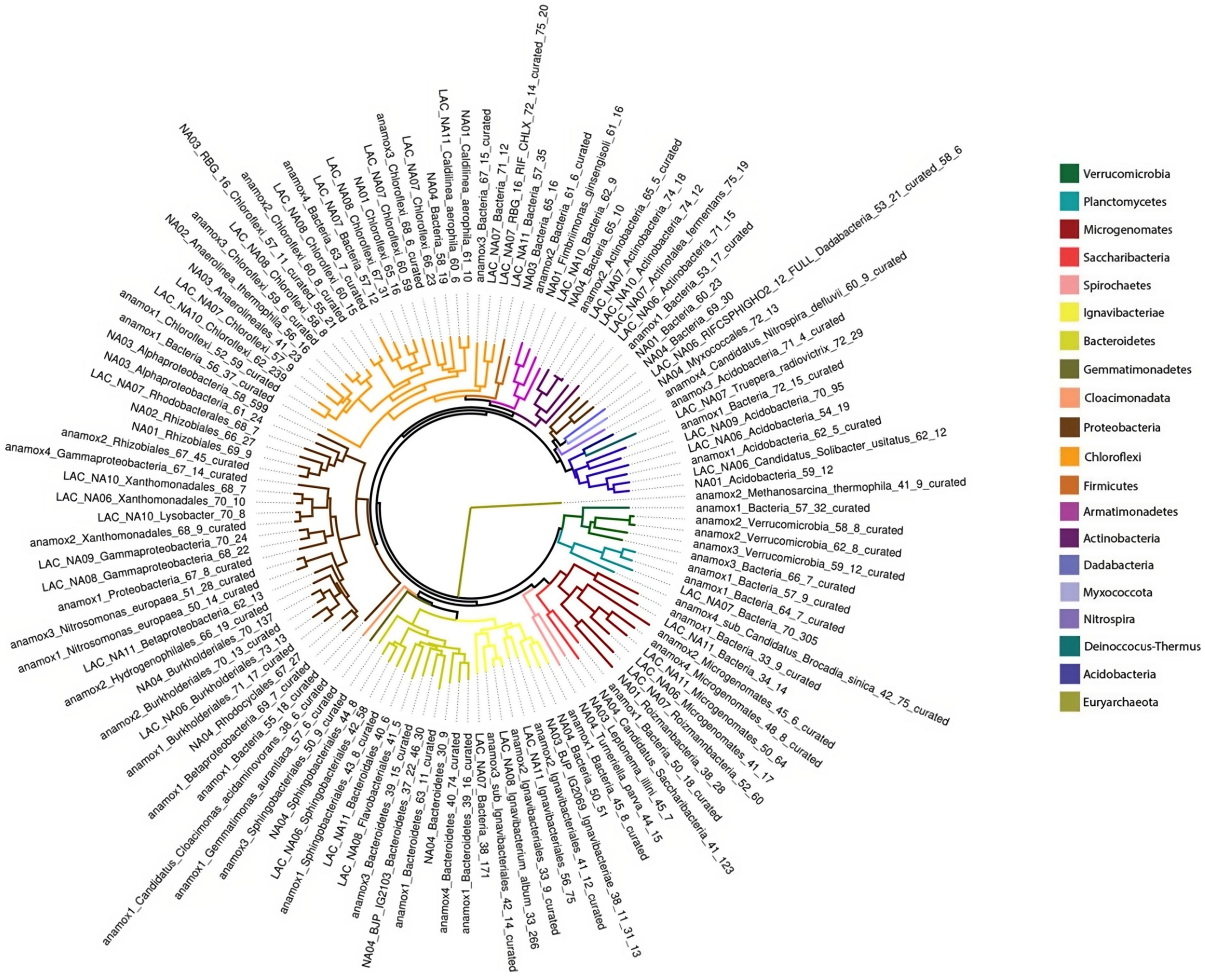
Maximum likelihood phylogenetic tree from concatenated ribosomal proteins of three metagenomic samples taken during the experiment.

After read quality control 342,892,526 reads were obtained from all three metagenome samples. Reads were assembled into Metagenome Assembled Genomes (MAG), resulting in 27,836 contigs with a median N50 of 2,138 bp. Contigs were binned to draft genomes resulting in 129 draft genomes accounting for 74.62% of quality filtered reads on average across all three samples (Supplementary Table S4). These MAGs represented 20 bacterial phyla and one archaeal phylum, as shown in Figure 4. The most abundant genomes based on coverage were affiliated with Planctomycetes, Proteobacteria, Ignavibacteriae, Chloroflexi, and the super-phylum Candidate Phyla Radiation (CPR). A full list of genomes is available in Supplementary Table S5. AMX1 (*Brocadia sinica*), the only anammox MAG recovered, accounted for 44.97% of all reads on average across all three metagenome samples. AMX1 had an average GC content of 42.29%, a genome size of 3.1 Mbp, and 2,859 open reading frames (ORFs) which was consistent with other previously reported anammox genomes for this strain (Oshiki et al., 2015; Feng et al., 2019).

### 3.4. Nitrogen cycle gene abundances

**Figure 5:**
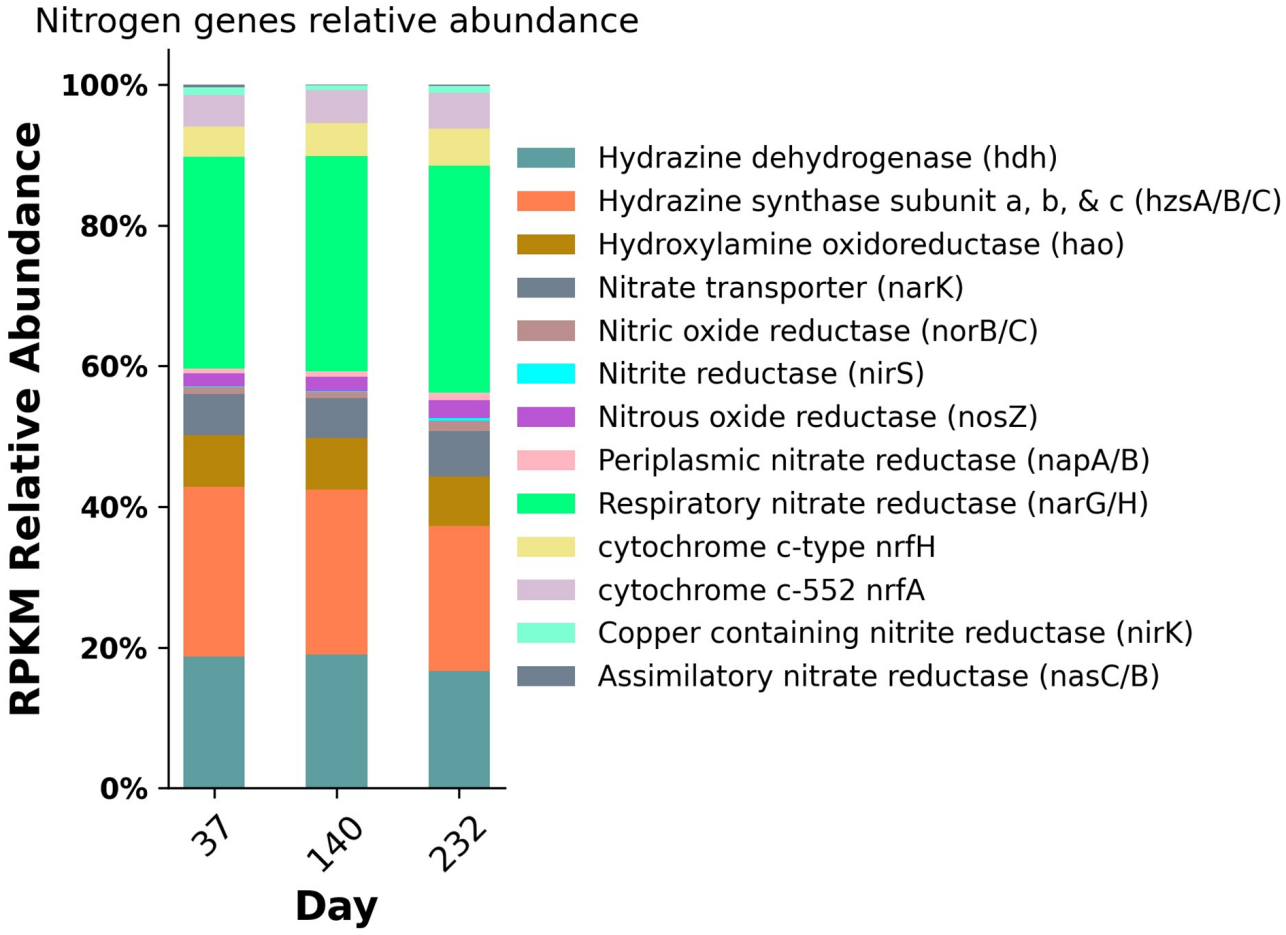
Nitrogen gene relative abundances from metagenomic sequencing from three time points during the experiment.

To assess the changes in abundance of nitrogen metabolic genes, the relative abundance of these genes was calculated for each metagenomic sampling timepoint (Figure 5). The metabolic genes with the highest abundance throughout the duration of the experiment included hydrazine dehydrogenase (*hdh*), hydrazine synthase (*hzsA/B/C*), hydroxylamine oxidoreductase (*hao*), respiratory nitrate reductase (*narGH*), nitrous oxide reductase (*nosZ*), and cytochrome c membrane associated nitrite reductases (*nrfAH*) with relative abundances ranging from 4.3 to 32.3%. For the purposes of this study, we are utilizing *nrfAH*, the gene encoding the one-step reduction of nitrite to ammonium as a proxy for DNRA. Likewise, we are employing *nirK* and *nirS*, both used for nitrite reduction to nitric oxide, as proxies for denitrification. The abundance of functional analogs *nirK* and *nirS*, were only 0.9 and 0.1%, respectively; however, the relative abundance of respiratory nitrate reductases *narGH* was 30.99% on average, indicating the wide prevalence of nitrate reduction capacity. The nitrogen gene relative abundances for samples from day 37 and 140 were nearly identical; by day 232, the relative abundance of *nrfAH* nitrite reductases and *narGH* nitrate reductases increased slightly and the relative abundance of anammox associated hydrazine synthase and hydrazine dehydrogenase decreased slightly (Figure 5). The taxonomic variability and relatively stable metabolic composition suggest high levels of functional redundancy amongst bacteria in the reactor.

**Figure 6:**
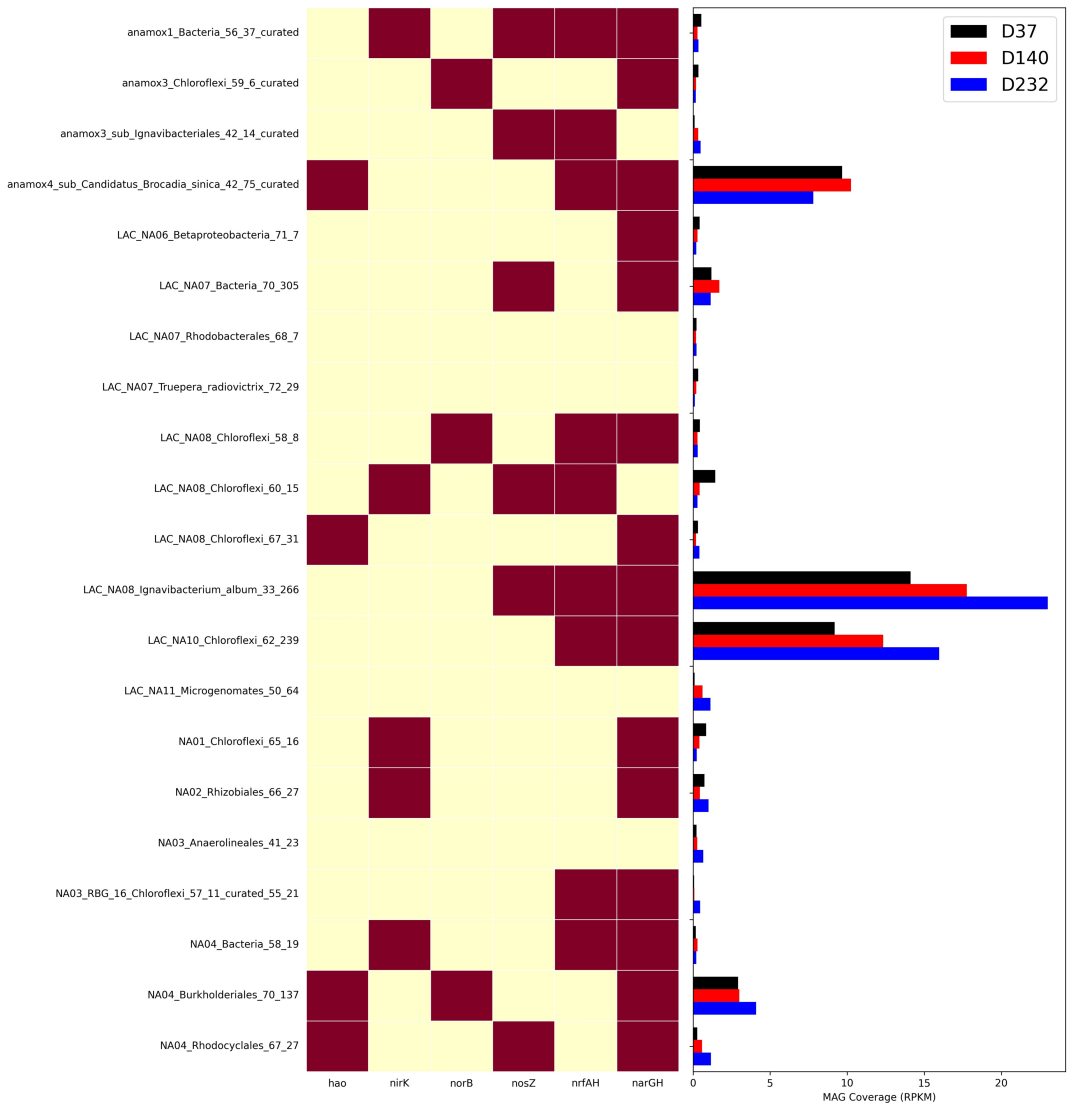
Heatmap showing presence of nitrogen genes in MAGs and abundance of MAGs from three timepoints with metagenome samples measured as RPKM.

To understand how different nitrogen metabolisms influenced microbial competition throughout the duration of the experiment, the abundance of key individual MAGs was calculated for each timepoint as shown in Figure 6. *narGH* nitrate reductases were the most abundant of any nitrogen metabolic gene and widely distributed amongst MAGs. The next most abundant nitrogen metabolism gene identified in key MAGs was *nrfAH*. The top 5 most abundant MAGs including AMX01 (*Brocadia*), IGN01 (*Ignavibacterium*), CFLX01 (Chloroflexi), PROT01 (Burkholderiales), and BAC01 all contained *narGH.* AMX01, CFLX01, and IGN01 also contained *nrfAH*. The abundance of AMX01 decreased from 9.67 RPKM on day 37 to 7.81 RPKM on day 232, while the abundances of CFLX01 and IGN01 increased from 14.11 and 9.18 RPKM on day 37 to 23.02 and 15.98 RPKM on day 232. Many of the MAGs that encoded for *narGH* also lacked the remaining genes necessary for the full denitrification pathway, which could result in the accumulation of nitrite. This is consistent with previously reported results (Speth et al., 2016) supporting the presence of a nitrite loop, in which nitrate is recycled back to anammox bacteria via partial denitrification of nitrate to nitrite (Kartal et al., 2011). Many genomes encoding for DNRA also encode genes for partial denitrification but for the purposes of our analysis, we assume these bacteria will carry out DNRA as the most energetically favorable pathway.

**Figure 7:**
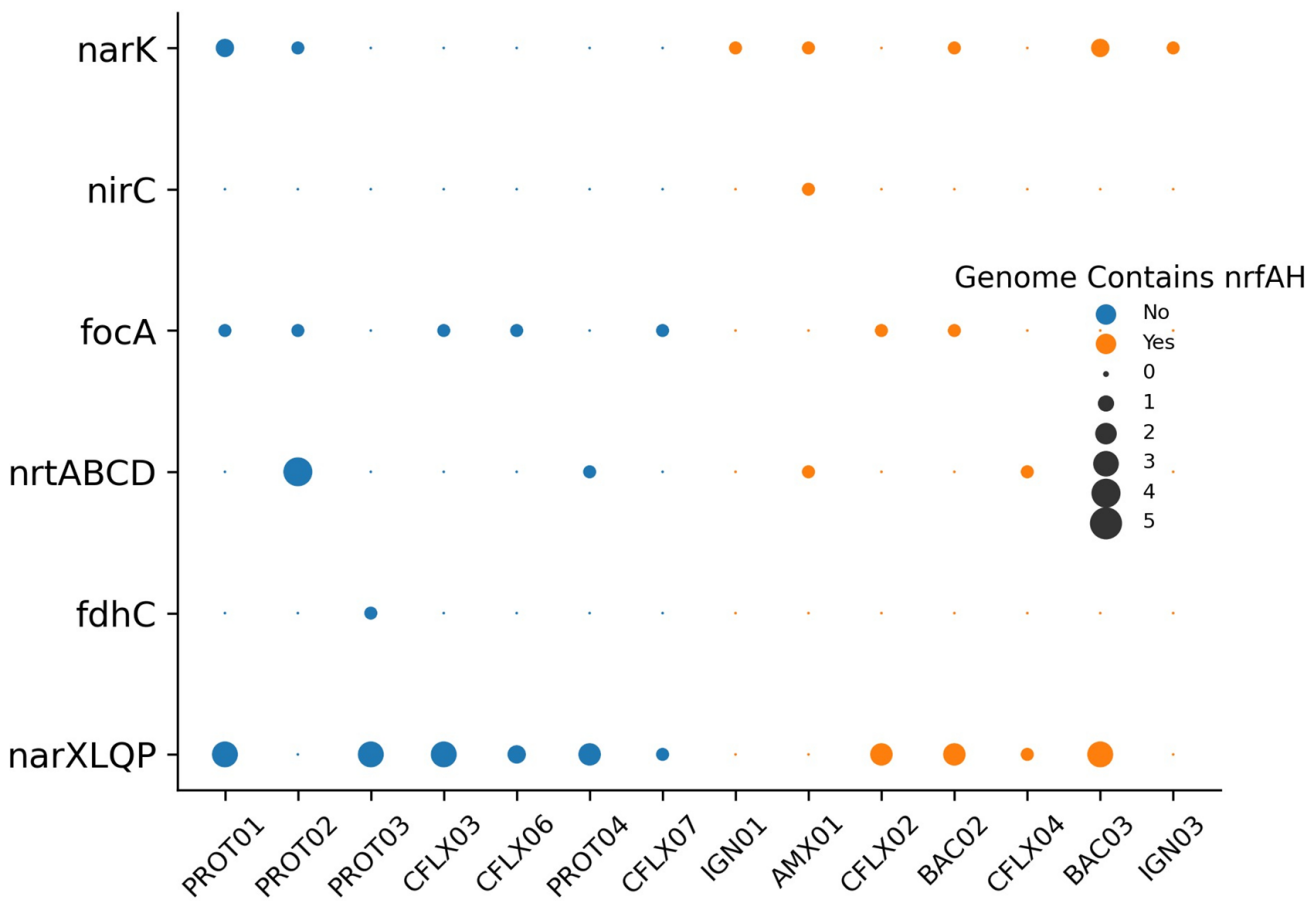
Putative nitrate/nitrite transporters and sensors encoded in MAGs.

Nitrogen transport and sensing genes, which can be used to infer substrate affinity, were identified in the top 14 most abundant MAGs by coverage (Figure 7), and all MAGs with genome coverage above 40% (Supplementary Figure S4). This analysis was performed to assess how effectively different organisms compete for various nitrogen substrates. Nitrate, a byproduct of anammox biosynthesis, is frequently abundant in anammox reactors, while nitrite is kept limiting to prevent nitrite toxicity. Therefore, the bacteria with the greatest affinity for nitrite will have a competitive advantage. Most genomes contained transporters belonging to the Nitrate/Nitrite Porter (NNP) family, including *narK*, *nrtP*, and *nasA* for nitrate and nitrite membrane transport (Moir and Wood, 2001). Consistent with previously reported anammox metagenomes (Ji et al., 2022; Wang et al., 2022), AMX01 contained Nitrate Transporter superfamily (NRT) nitrate/nitrite transporters, the high affinity nitrate/nitrite transporter *nrtB*, and a polytopic membrane transporter specifically for nitrite, *nirC.* Several genomes, excluding those of anammox bacteria, also encoded genes for putative formate-nitrite transporters from the formate-nitrate transporter (FNT) superfamily, including formate channel *focA* and formate permease *fdhC*. Previous research has shown that some denitrifying bacteria have a higher affinity for nitrite than nitrate (Kraft et al., 2014). This was supported by the presence of FNT transporters in a higher proportion of MAGs without *nrfAH* than those with these nitrate-metabolizing genes. The majority of genomes also encoded nitrate/nitrite sensor protein complexes *narX/L* and *nar Q/P*. These genes, which are often colocated on the *nar* operon, have been demonstrated to regulate expression of nitrate/nitrite reductases as well as other respiratory proteins (Browning et al., 2006).

### 3.5. Microbial community dynamics

**Figure 8:**
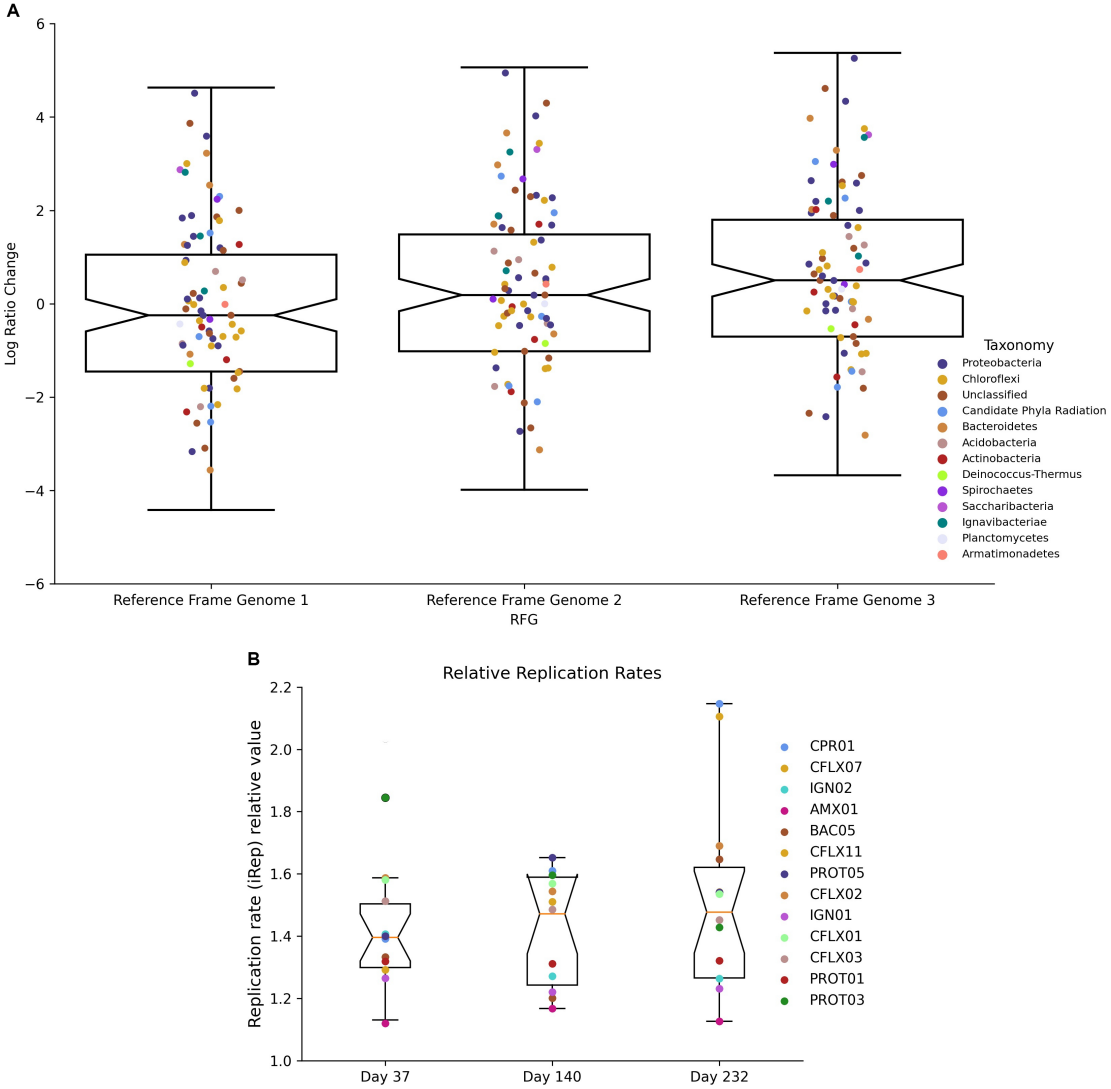
**(A)** Log ratio changes between D37 and D232 using different genomes as reference frames. **(B)** Replication rate values on D37, D140, and D232.

Log-ratio (LR) changes between day 232 and day 37 were calculated using each MAG’s coverage normalized to three reference genomes (Figure 8A). Of the 50 MAGs that experienced LR changes above the upper 95% confidence interval (CI) (Supplementary Table S7), 25 had RPKM abundances lower than the 95% CI (Supplementary Table S9) and 32 contained *narGH*, 15 contained *nirKS*, and 17 contained *nrfAH*. Of the 57 MAGs that experienced LR changes below the lower 95% CI, 36 had RPKM abundances lower than the lower 95% CI, and 35 contained *narGH*, 21 contained *nirKS*, and 13 contained *nrfAH*. Significant log-ratio changes in coverage did not have a strong correlation with taxonomy (Supplementary Table S6).

iRep values, which estimate each MAG’s replication rate, ranged between 1.12 and 2.29 (Figure 8B). As expected, *Brocadia* had the lowest replication rates at all three time points with an average of 1.14. CFLX13 (Chloroflexi) had the highest replication rates with an average of 2.15. PROT03 (Rhodocyclales) experienced the most substantial decrease in replication rates during the course of the experiment, falling from 1.85 at day 37 to 1.43 at day 232 (a 23% decrease); CPR01 experienced the most significant increase, from 1.39 on day 37 to 2.15 on day 232 (a 55% increase). With the exception of CPR01, all of the MAGs that demonstrated increased replication rates between time points had negative log-ratio changes between day 37 and 232. This observation could be an indication of higher mortality rates suggestive of r-type strategists, or organisms well adapted to optimize growth during unstable environmental conditions. These types of organisms have shorter life cycles but very high reproduction rates associated with low efficiency substrate utilization (Grime, 1977; Andrews and Harris, 1986; Ho et al., 2017).

### 3.6. Taxon abundance and reactor operational parameters

**Figure 9:**
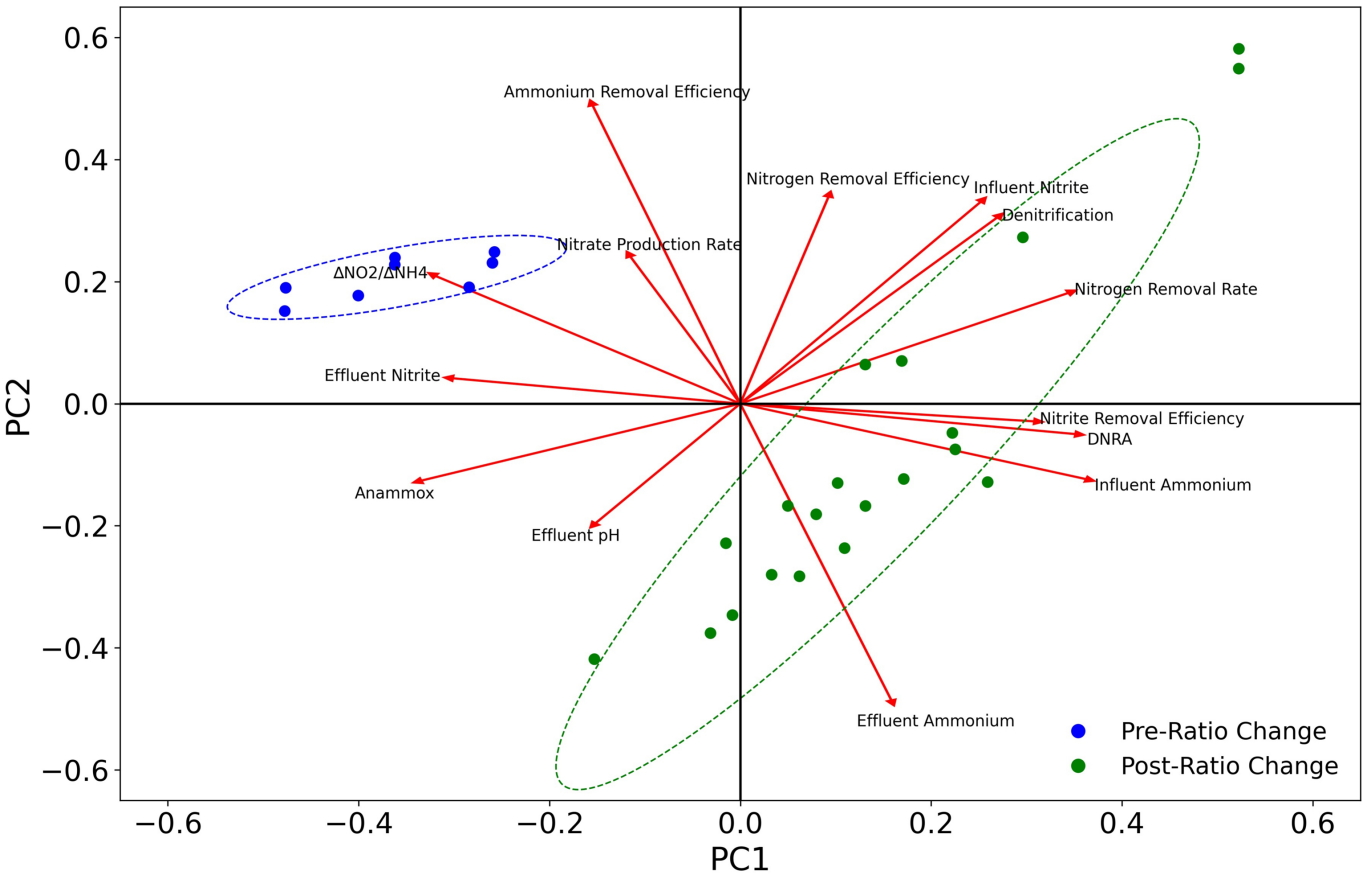
PCA plot of changes in taxa abundance for anammox, denitrification, and DNRA with reactor operational parameters. Ellipses represent 95% confidence intervals.

To evaluate the effects of different nitrogen pathways on reactor performance, PCA analysis was conducted for the abundance of anammox, *nirKS* (denitrifying), and *nrfAH* (DNRA) associated taxa (Figure 9). The abundance of anammox bacteria is positively correlated with ammonium removal efficiency and the nitrate production rate, indicating the anammox bacteria are primarily responsible for ammonium removal and nitrate production as expected. The abundance of DNRA and denitrifying bacteria is positively correlated with nitrogen removal efficiency, nitrite removal efficiency, and the nitrogen removal rate, and negatively correlated with the ammonium removal efficiency, nitrate production rate, and effluent nitrite concentrations. However, the abundance of DNRA bacteria is more strongly associated with nitrite removal efficiency than denitrification. These results are consistent with the performance of the MBR throughout the course of the experiment, as the abundance of anammox bacteria decreased after the ratio change on day 89, while the ammonium removal efficiency decreased as well. Nevertheless, the overall nitrogen removal rate and nitrogen removal efficiency increased along with the increased growth of DNRA bacteria.

## 4. Discussion

### 4.1. Microbial community shifts in reactor

While previous studies have examined the performance of anammox reactors under variable influent loading ratios, analyses of the effects on the microbial community are limited. After the introduction of decreased NH_4_^+^:NO_2_^−^ ratios, we observed an increase in the microbial diversity of the system. Of the 317 genera identified through amplicon sequencing, 36% increased by at least one order of magnitude after the ratio change, and the alpha diversity also increased steadily. This increased diversity may correspond to increased availability of ammonium as a growth-limiting nitrogen source. Of the 129 MAGs identified, only 25 contained canonical assimilatory nitrate/nitrite reductases to utilize nitrate or nitrite as nitrogen sources; this suggests that most of the microbial community may require ammonium as a fixed nitrogen source for cell growth. During the initial nitrogen loading ratio, effluent ammonium concentrations remained low, within the range of 0–0.143 mM. Anammox bacteria are known to have high affinities for ammonium, as evidenced by the presence of multiple ammonium transporters in their genomes (Hu et al., 2012; Kartal et al., 2012; Van Niftrik and Jetten, 2012). This gives anammox microbes a competitive advantage for ammonium uptake, even at low concentrations. When the ratio of nitrogen substrates changed, the low fixed nitrite concentration would have limited ammonium oxidation, leading to an increased concentration of ammonium in the reactor, in excess of what anammox bacteria could use. This resulted in increased reactor ammonium concentrations (Figure 1), which may have supported the growth of other organisms with lower affinities for ammonium uptake to support biosynthesis.

Increased microbial diversity could also be explained by the interaction of diversifying stochastic drift constrained by deterministic homogenous selection. Based on theory supporting these processes, a microbial community will trend towards functional homogeneity during static environmental conditions (homogenous selection), but will also undergo variable changes in composition due to weak selection pressures (drift) (Stegen et al., 2015; Zhou and Ning, 2017). In anammox reactors, these processes would result in the selection of bacteria well-adapted to the conditions created by reactor operational parameters and the dominance of anammox bacteria, independent of taxonomy, as shown through the log-ratio changes (Figure 8A). However, when the reactor maintains these environmental conditions for extended periods of time, weak selection can promote fluctuations in the microbial community composition. Throughout the duration of the experiment, the reactor maintained relatively stable performance, except for effluent ammonium concentrations rising post-ratio change. Despite this consistency, populations of bacterial taxa from Ignavibacteriae, Chloroflexi, and Bacteroidetes still fluctuated. This is similar to results presented by Ya et al. (2023) where increased abundance of Chloroflexi and Proteobacteria was observed despite stable reactor operation. Functional redundancy amongst these bacterial groups could contribute to their increased abundance, despite the lack of any operational or natural perturbations. This hypothesis, combined with the sustained high activity of anammox bacteria despite low abundance, could provide an explanation for the maintenance of efficient bioreactor performance (>85% nitrogen removal) despite significant changes to the microbial community.

### 4.2. Synergistic interactions between DNRA and anammox

After the influent NH_4_^+^:NO_2_^−^ ratio change, we observed increased abundance of bacteria capable of performing DNRA. Amplicon sequencing and shotgun metagenomic sequencing revealed increased abundance of bacteria belonging to Ignavibacteriae, Chloroflexi, and Bacteroidetes, phyla previously associated with DNRA in anammox reactors (Wang et al., 2019; Keren et al., 2020; Bryson et al., 2022). While there is not sufficient evidence to conclude that the ratio change directly led to increased abundance of DNRA bacteria in this reactor system, the co-occurrence of this trend with stable reactor operation is an intriguing observation worth consideration. Previous studies have reported the presence and increased replication of DNRA bacteria to coincide with decreased reactor performance (Keren et al., 2020). However, DNRA bacteria have also been shown to form symbiotic interactions with anammox bacteria, promoting robust reactor performance (Li et al., 2020; Sheng et al., 2021). Many anammox species can also perform DNRA using simple organic acids as substrates (Castro-Barros et al., 2017), further complicating the characterization of the interplay between these metabolisms. Zhou et al. (2023) reported very similar results to those observed in this work, demonstrating increased abundance of DNRA bacteria with improved nitrogen removal efficiency (NRE) at lower NH_4_^+^:NO_2_^−^ ratios, but deleterious effects on NRE at higher NH_4_^+^:NO_2_^−^(Zhou et al., 2023). The results from that study illustrate the delicate balance between anammox and DNRA, and how the complexity of these interactions is impacted by influent loading rates, organic carbon concentrations, and anammox species niche differentiation.

The observation of increased abundance of bacteria utilizing DNRA post-ratio change could be explained using a few key considerations. Previous research on the competitive balance between DNRA and denitrification suggests that DNRA is generally favored over denitrification at higher C/N ratios (van den Berg et al., 2016). This observation has also been purported to apply for both nitrate and nitrite and under dual limitation conditions when both the supply of electron donor and electron acceptor are limited in the environment (van den Berg et al., 2017a). Organic carbon concentrations, estimated from the biodegradable fraction of biomass measured through MLVSS (Supplementary Figure S3) remained substantially higher than effluent nitrate and nitrite concentrations throughout the duration of the experiment indicating conditions favorable for DNRA. From day 0 up until day 89 when the influent NH_4_^+^:NO_2_^−^ ratio was 1:1.32, anammox bacteria, DNRA bacteria, and denitrifiers actively competed for nitrite and anammox bacteria were able to sustain relative dominance likely due to a higher affinity for nitrite as evidenced by multiple nitrite transporters (Figure 7). However, on day 89 when the NH_4_^+^:NO_2_^−^ ratio was shifted to 1:1.1, the influent ammonium concentration was increased, and this would have required more nitrite to undergo the anammox reaction to completely oxidize ammonium to dinitrogen gas. Lowering the NH_4_^+^:NO_2_^−^ ratio could have intensified the competition for nitrite, and this increased competition could select for bacteria that are capable of utilizing already limited substrates more efficiently. From a thermodynamic perspective, the theoretical amount of energy produced per mole of nitrite for DNRA and anammox is comparable (Castro-Barros et al., 2017). However, the yield of biomass produced per mole of nitrite through DNRA is effectively higher than through anammox, and bacterial growth rates are also significantly higher under DNRA (Figure 8B). Thus, when the ratio shift occurred and nitrite limitation was intensified, DNRA bacteria could have been poised to proliferate because of their ability to grow and biosynthesize more efficiently. It is important to consider that anammox bacteria also convert nitrite to nitrate to generate reducing equivalents for carbon fixation and biomass production (Strous et al., 2006). Additional competition for nitrite could lead to anammox bacteria diverting more nitrite towards energy generation than carbon fixation. This could result in decreased biomass production, which could offer a possible explanation for the decrease in cell abundance observed throughout the duration of the experiment. This explanation neglects the capability of anammox bacteria to utilize partial DNRA to convert nitrate back to nitrite to use for the anammox reaction. However, this reaction has only explicitly been identified to support the oxidation of volatile fatty acids (VFAs) (Castro-Barros et al., 2017) and is uncharacterized for alternative carbon substrates.

Reactor configuration and biomass growth type adds another dimension of complexity to the dynamics between anammox, DNRA, and denitrifying bacteria. Bryson et al. (2022) reported higher abundance of Ignavibacteriae and Phycisphaerae with *nrfAH* genes used for DNRA in two stage anammox configurations as compared with one stage configurations, citing a negative selection pressure on facultatively aerobic denitrifiers in a strictly anaerobic environment (Bryson et al., 2022). Our findings are consistent with those observations given that the strict anaerobic conditions provide a niche for fermentative bacteria (Gonzalez-Gil et al., 2015; Speth et al., 2016; Bhattacharjee et al., 2017). These observations could provide insight into the conditions that favor DNRA in anammox systems. Many of the bacteria with *nrfAH* could be coupling DNRA to the fermentation of extracellular amino acids and exogenous carbon substrates. This reaction is more bioenergetically favorable than pure fermentation (Kraft et al., 2011), and could be even more competitive than alternative nitrogen pathways when the carbon substrate is more reduced. IGN01 and IGN03 both contain genes encoding for acetate kinase, and CFLX01 encodes genes for acetate ligase, suggesting that these strains have the ability to ferment acetate or propionate. This would be especially advantageous under the conditions found in the MBR where the concentrations of fermentable substrates, such as extracellular amino acids and polysaccharides, are likely much higher than non-fermentable substrates such as acetate, ethanol, or methanol. Fermentable sugars such as D-Arabinose, D-Ribose, and D-Mannose and sialic acids such as neuraminic acid, commonly found in anammox extracellular polysaccharides (Yin et al., 2015; Boleij et al., 2020; Wang et al., 2020), have also been shown to selectively enrich DNRA bacteria over denitrifiers (Carlson et al., 2020). When the ratio shift occurred, and the competition for nitrite intensified, the ability to couple nitrite reduction to fermentation of highly reduced carbon substrates could have also contributed to the enrichment of DNRA. Thus, the type of carbon source and the oxidative conditions in anammox systems also has a substantial impact on the competitive balance between anammox, denitrifiers, and DNRA bacteria.

**Figure 10:**
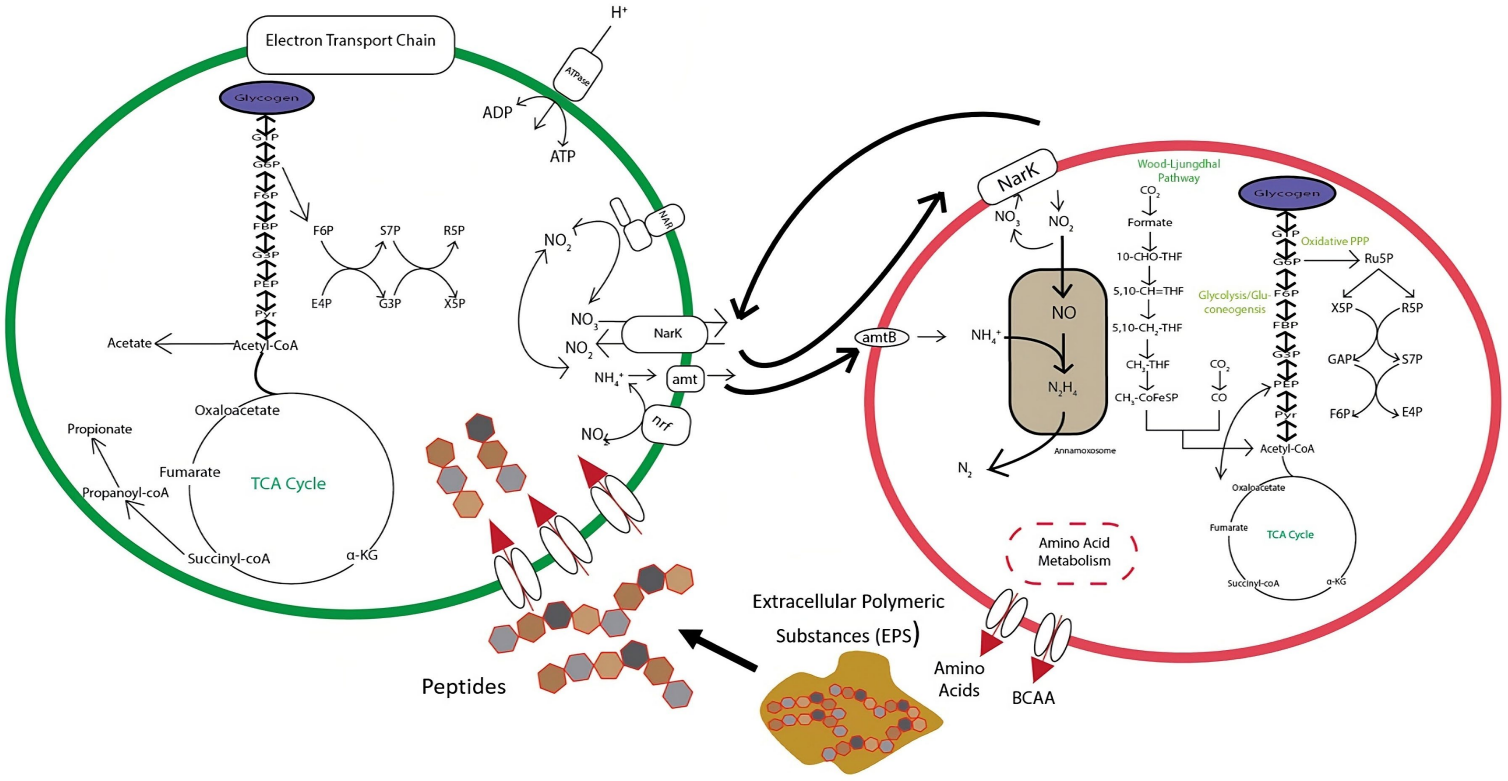
Conceptual diagram of metabolic exchanges occurring between anammox and DNRA bacteria within the tested bioreactor.

The balance between DNRA and anammox is predicated on an exchange of nitrate, ammonium, and organic carbon substrates as shown in Figure 10. DNRA bacteria are thought to utilize substrates derived from extracellular polymeric substances (EPS) produced by anammox bacteria (Keren et al., 2020), which would necessitate a symbiotic relationship between these two pathways. DNRA can provide anammox bacteria with nitrite through the reduction of nitrate via *nar* and *nap* nitrate reductases, or ammonium through the reduction of nitrite with *nrfAH* nitrite reductases. This steady flow of material exchanges can result in enhanced nitrogen removal in a variety of ecosystems (Ahmad et al., 2021; Sheng et al., 2021). Despite this, DNRA can still actively compete with anammox for nitrite, and even potentially destabilize aggregates through overconsumption of EPS, leading to anammox cell death (Keren et al., 2020). This tipping point is a pivotal junction to distinguish in order to optimize reactor efficiency and resiliency. It is also important to evaluate how this relationship changes at more extreme NH_4_^+^:NO_2_^−^ ratios (<1:1.1 and >1:1.3). Thus, further research is needed to identify and parameterize the equilibrium of this dynamic.

### 4.3. Reactor performance implications

Anammox reactors are known to be sensitive to operational conditions and are susceptible to destabilizations after perturbation. These systems are particularly sensitive to nitrite fluctuations (Bettazzi et al., 2010; Puyol et al., 2014). The formation of synergistic relationships between anammox and DNRA can help to alleviate the deleterious effects of nitrite inhibition by keeping nitrite concentrations low. These interactions have been demonstrated to assist in the recovery of anammox reactor performance following nitrite inhibition (Qiao et al., 2022; Zhou et al., 2023), and to help stimulate the recovery of anammox bacteria from dormancy (Zhu et al., 2019). Promoting synergy between anammox and DNRA bacteria could be even more important at higher nitrogen loading rates, which cause the reactor to be more susceptible to nitrite inhibition (Tang et al., 2010). Throughout the duration of this study, nitrite concentrations rarely rose above detectable levels in the effluent, which can be attributed to the abundance of excess ammonium for the anammox reaction (1 mole of NH_4_^+^ requires 1.32 moles of NO_2_^−^ to be fully oxidized) but also to the reduction of nitrite to ammonium by DNRA bacteria and, to a lesser extent, nitrite reduction to dinitrogen gas by denitrifying bacteria. The abundance of DNRA bacteria was also positively correlated with the nitrite removal efficiency, nitrogen removal efficiency, and nitrogen removal rates (Figure 9). These results provide evidence for the positive contributions of DNRA towards robust reactor performance. The interactions between anammox and DNRA bacteria can be enhanced by organic carbon amendments to encourage the growth of DNRA microbes, which has been previously reported to have beneficial effects on reactor performance (Jenni et al., 2014; Zhang et al., 2021; Fan et al., 2022). Taking advantage of the synergy between anammox and DNRA could be especially advantageous during reactor startup, where anammox activity is vulnerable to performance disruptions due to nitrite inhibition. These interactions could also be leveraged during periods of elevated nitrite concentrations by dosing with organic carbon to stimulate the growth of DNRA bacteria.

## 5. Conclusion

In a single-stage anammox MBR, adjusting influent NH_4_^+^:NO_2_^−^ from the conventional ratio of 1:1.32 to 1:1.1 led to a significant change in the microbial community. Despite relatively minor changes in total nitrogen removal efficiency (85.26 ± 0.01% pre-ratio change vs. 85.833 ± 0.002% post-ratio change), the relative abundance of anammox bacteria in the system decreased from 77.19 to 12.24% by 16S rRNA amplicon sequencing. This coincided with the growth of bacteria capable of performing DNRA; the phyla Ignavibacteriae and Chloroflexi increased from 7.73 and 4.57%, respectively, to 23.36 and 38.22%. These results demonstrate the positive effects of a stable dynamic between anammox and DNRA, which can result in robust reactor performance and enhanced nitrogen removal.

## Data availability statement

The datasets presented in this study can be found in online repositories. The names of the repository/repositories and accession number(s) can be found in the article/Supplementary material.

## Author contributions

EA and JL supervised the study. EA, JL, and LA-C designed the study. EA and JL maintained the bioreactor. CW analyzed the bioreactor performance, 16S rRNA gene data, metagenomics data, and wrote the manuscript. SS contributed to data analysis and visualization. RK contributed to metagenomic data analysis. LJ, KY, and WZ assisted with study design and manuscript revision. All authors read the manuscript and contributed with their input.

## Funding

This research was supported by the National Natural Science Foundation of China (Nos. 51709005 and 51708362), and the National Science Foundation through the Engineering Research Center for ReInventing the Nation’s Water Infrastructure (ReNUWIt) ECC-1028962. This material is also based upon work supported by the National Science Foundation Graduate Research Fellowship under grant no. DGE 1106400.

## Conflict of interest

Author JL was employed by CDM Smith.

The remaining authors declare that the research was conducted in the absence of any commercial or financial relationships that could be construed as a potential conflict of interest.

## Publisher’s note

All claims expressed in this article are solely those of the authors and do not necessarily represent those of their affiliated organizations, or those of the publisher, the editors and the reviewers. Any product that may be evaluated in this article, or claim that may be made by its manufacturer, is not guaranteed or endorsed by the publisher.

## Author disclaimer

Any opinion, findings, and conclusions or recommendations expressed in this material are those of the authors and do not necessarily reflect the views of the National Science Foundation.

## Footnotes

^1^https://www.r-project.org/

^2^https://www.rstudio.com/
