# Supplementary material for: Synergistic interactions between anammox and dissimilatory nitrate reducing bacteria sustains reactor performance across variable nitrogen loading ratios
Source: Front Microbiol. 2023 Aug 9;14:1243410. doi: 10.3389/fmicb.2023.1243410 (PMC10450351; doi:10.3389/fmicb.2023.1243410)
Supplement: Supplementary file 1 [file Data_Sheet_1.docx]

| **Constituent** | **Concentration** | **Unit** |
| --- | --- | --- |
| NH_4_HCO_3_ | 0-2000 | mg-N/L |
| NaNO_2_ | 0-2000 | mg-N/L |
| NaCl | 1000 | mg/L |
| MgCl_2_·6H_2_O | 500 | mg/L |
| KH_2_PO_4_ | 27.2 | mg/L |
| KCl | 10 | mg/L |
| CaCl_2_·2H_2_O | 180 | mg/L |
| NaHCO_3_ | 500 | mg/L |
| FeCl_2_·4H_2_O | 17.89 | mg/L |
| CoCl_2_·6H_2_O | 0.24 | mg/L |
| MnCl_2_·4H_2_O | 0.99 | mg/L |
| ZnCl_2_ | 0.2 | mg/L |
| H_3_BO_3_ | 0.014 | mg/L |
| Na_2_MoO_4_·2H_2_O | 0.22 | mg/L |
| NiCl_2_·6H_2_O | 0.19 | mg/L |
| CuCl_2_·2H_2_O | 0.17 | mg/L |
| Na_2_SeO_3_·5H_2_O | 0.16 | mg/L |
| pH | 6.8-7.0 |  |

SI Table 1: MBR Media Composition


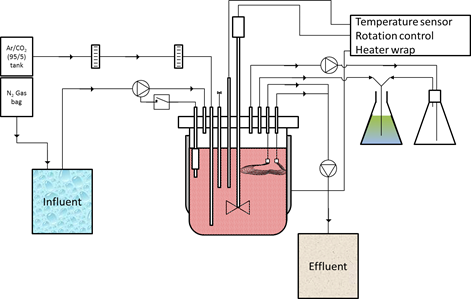


SI Figure 1: Mechanical Sketch of MBR

| **Operational Parameter** | **Setting** | **Unit** |
| --- | --- | --- |
| Gas flow rate (Ar/CO_2_:95/5) | 0.2 | LPM |
| Temperature | 37 | ℃ |
| Mixing | 200 | RPM |
| HRT | 18-24 | hr |
| SRT | 50-60 | days |
| Volume | 1 | L |

SI Table 2: MBR Operational Parameters


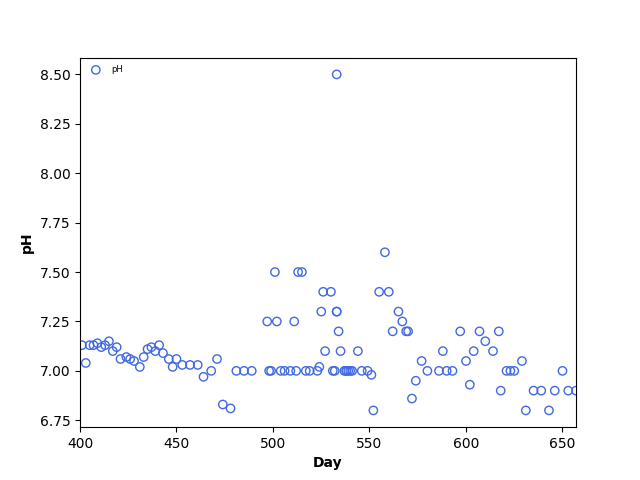


SI Figure 2: MBR Effluent pH


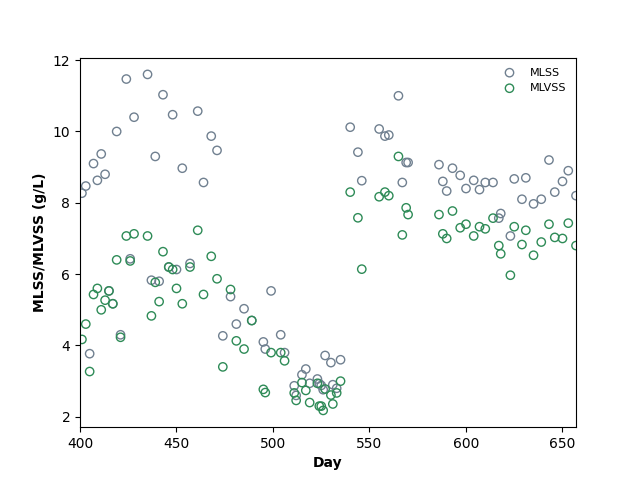


SI Figure 3: MLSS/MLVSS Measurements

| Day | Chao | Shannon | Simpson |
| --- | --- | --- | --- |
| 16 | 363.5769 | 1.522672 | 0.496445 |
| 17 | 500 | 1.623997 | 0.548803 |
| 18 | 276 | 1.211802 | 0.402098 |
| 21 | 671.3125 | 2.368713 | 0.767801 |
| 33 | 573.6471 | 2.053709 | 0.689854 |
| 41 | 466.3684 | 2.089925 | 0.709396 |
| 43 | 269.7143 | 2.368648 | 0.781668 |
| 46 | 448.9545 | 2.387704 | 0.780954 |
| 99 | 718.1429 | 2.223641 | 0.743966 |
| 117 | 577.1714 | 1.714986 | 0.640657 |
| 130 | 581.9024 | 1.948363 | 0.689643 |
| 131 | 518 | 1.856925 | 0.662794 |
| 132 | 553.8571 | 1.873487 | 0.6956 |
| 134 | 642.025 | 2.170183 | 0.780811 |
| 138 | 724 | 2.178913 | 0.778882 |
| 140 | 778.35 | 2.727573 | 0.858208 |
| 149 | 942.2885 | 2.655922 | 0.852828 |
| 152 | 869.1111 | 2.701284 | 0.825935 |
| 159 | 811.2128 | 2.653657 | 0.852464 |
| 168 | 558.9268 | 2.201301 | 0.744807 |
| 181 | 955.5714 | 2.651988 | 0.854377 |
| 188 | 1025.325 | 2.749838 | 0.866492 |
| 194 | 819.15 | 2.502905 | 0.82332 |
| 203 | 787.0857 | 2.578343 | 0.848444 |
| 211 | 772.0612 | 2.693486 | 0.873011 |
| 217 | 991.626 | 2.93486 | 0.891122 |
| 245 | 973.9608 | 3.012237 | 0.899178 |
| 252 | 1167.917 | 2.97098 | 0.897576 |

SI Table 3: 16s rRNA Alpha Diversity Indices

SI Table 4: Metagenome alignment statistics


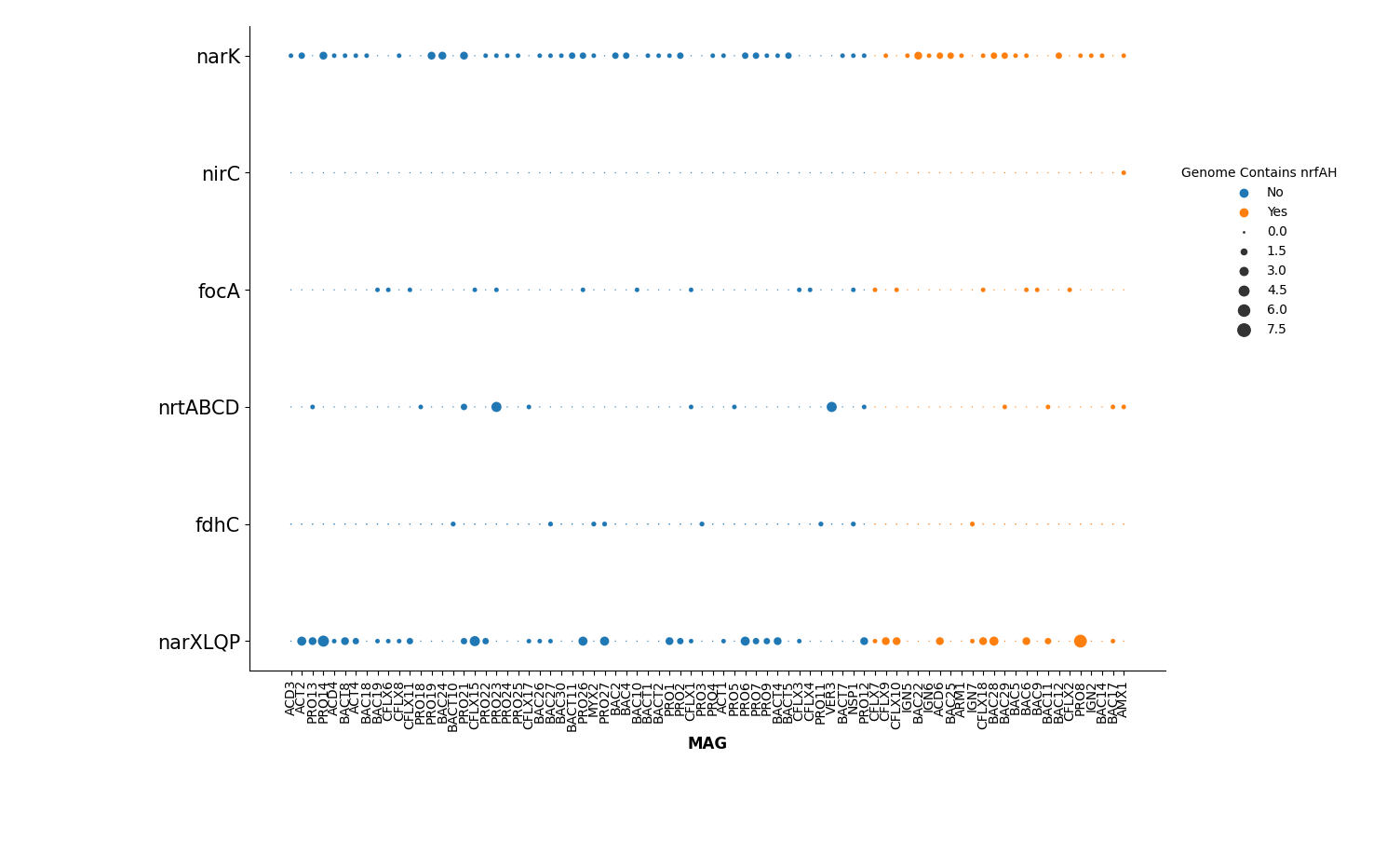


SI Figure 4: Nitrogen transport and sensing genes for MAGs with at least 40% genome coverage

SI Table 5: Metagenome assembled genome (MAG) statistics

| **Genome** | **Genome code (ggkbase)** | **Phyla** | **Genome length (bp)** | **GC%** | **Contigs** | **ORFs** | **Completeness (%)** |
| --- | --- | --- | --- | --- | --- | --- | --- |
| ACD01 | LAC_NA09_Acidobacteria_70_95 | Acidobacteria | 3,730,000 | 69.99% | 45 | 3263 | 92.74% |
| ACD02 | NA01_Acidobacteria_59_12 | Acidobacteria | 5,590,000 | 59.15% | 159 | 4789 | 92.98% |
| ACD03 | LAC_NA06_Candidatus_Solibacter_usitatus_62_12 | Acidobacteria | 4,520,000 | 62.17% | 166 | 3970 | 84.35% |
| ACD04 | anamox3_Acidobacteria_71_4_curated | Acidobacteria | 1,330,000 | 69.62% | 772 | 1759 | 35.00% |
| ACD05 | anamox1_Acidobacteria_62_5_curated | Acidobacteria | 3,110,000 | 61.59% | 565 | 3251 | 69.92% |
| ACD06 | LAC_NA06_Acidobacteria_54_19 | Acidobacteria | 2,710,000 | 54.24% | 26 | 2505 | 94.02% |
| ACT01 | LAC_NA10_Actinobacteria_74_12 | Actinobacteria | 2,540,000 | 73.88% | 170 | 2460 | 96.24% |
| ACT02 | LAC_NA07_Actinobacteria_74_18 | Actinobacteria | 1,210,000 | 73.80% | 141 | 1247 | 58.54% |
| ACT03 | anamox2_Actinobacteria_65_5_curated | Actinobacteria | 2,800,000 | 63.61% | 662 | 3147 | 66.92% |
| ACT04 | LAC_NA06_Actinobacteria_71_15 | Actinobacteria | 3,180,000 | 70.59% | 57 | 3196 | 95.83% |
| ACT05 | LAC_NA07_Actinotalea_fermentans_75_19 | Actinobacteria | 2,910,000 | 75.44% | 31 | 2757 | 95.95% |
| AMX01 | anamox4_sub_Candidatus_Brocadia_sinica_42_75_curated | Planctomycetes | 3,110,000 | 42.29% | 64 | 2859 | 100% |
| ARC01 | anamox2_Methanosarcina_thermophila_41_9_curated | Euryarchaeota | 2,960,000 | 41.19% | 82 | 2703 | 97.85% |
| ARM01 | NA01_Fimbriimonas_ginsengisoli_61_16 | Armatimonadetes | 2,770,000 | 60.97% | 47 | 2570 | 93.98% |
| BAC01 | LAC_NA07_Bacteria_70_305 | Bacteria | 2,750,000 | 70.12% | 54 | 2386 | 90.91% |
| BAC02 | anamox1_Bacteria_56_37_curated | Bacteria | 4,970,000 | 55.88% | 43 | 4404 | 91.52% |
| BAC03 | NA04_Bacteria_58_19 | Bacteria | 7,560,000 | 57.56% | 112 | 6122 | 97.27% |
| BAC04 | LAC_NA07_Bacteria_38_171 | Bacteria | 2,390,000 | 37.56% | 23 | 2128 | 95.08% |
| BAC05 | LAC_NA07_Bacteria_57_12 | Bacteria | 5,860,000 | 57.26% | 271 | 5345 | 92.73% |
| BAC06 | anamox2_Bacteria_68_6_curated | Bacteria | 2,290,000 | 67.96% | 460 | 2621 | 60.64% |
| BAC07 | anamox4_Bacteria_63_7_curated | Bacteria | 3,450,000 | 62.64% | 351 | 3276 | 87.27% |
| BAC08 | NA04_Bacteria_65_10 | Bacteria | 4,100,000 | 64.60% | 238 | 3590 | 91.67% |
| BAC09 | LAC_NA07_Bacteria_71_12 | Bacteria | 2,760,000 | 71.10% | 206 | 2842 | 84.60% |
| BAC10 | NA04_Bacteria_69_30 | Bacteria | 4,020,000 | 69.42% | 30 | 3566 | 93.33% |
| BAC11 | NA01_Bacteria_60_23 | Bacteria | 3,780,000 | 60.01% | 48 | 3266 | 94.95% |
| BAC12 | LAC_NA10_Bacteria_62_9 | Bacteria | 3,000,000 | 62.13% | 264 | 2819 | 87.04% |
| BAC13 | LAC_NA11_Bacteria_34_14 | Bacteria | 863,630 | 34.10% | 17 | 886 | 79.15% |
| BAC14 | NA04_Bacteria_50_51 | Bacteria | 2,400,000 | 49.62% | 31 | 1990 | 94.81% |
| BAC15 | anamox3_Bacteria_67_15_curated | Bacteria | 2,770,000 | 66.48% | 144 | 2980 | 90.76% |
| BAC16 | anamox1__Bacteria_33_9_curated | Bacteria | 775,490 | 32.38% | 45 | 793 | 62.09% |
| BAC18 | anamox3_Bacteria_66_7_curated | Bacteria | 2,270,000 | 65.35% | 286 | 2150 | 81.93% |
| BAC19 | anamox1_Bacteria_64_7_curated | Bacteria | 3,990,000 | 63.42% | 306 | 3679 | 89.37% |
| BAC20 | anamox1_Bacteria_65_5_curated | Bacteria | 2,090,000 | 64.54% | 434 | 2237 | 52.01% |
| BAC21 | anamox2_Bacteria_61_6_curated | Bacteria | 2,420,000 | 60.87% | 385 | 2646 | 65.68% |
| BAC22 | LAC_NA11_Bacteria_57_35 | Bacteria | 6,060,000 | 56.96% | 90 | 5339 | 95.73% |
| BAC23 | anamox1_Bacteria_45_8_curated | Bacteria | 2,580,000 | 45.00% | 166 | 2221 | 89.32% |
| BAC24 | anamox1_Bacteria_57_9_curated | Bacteria | 3,470,000 | 57.28% | 74 | 3012 | 91.27% |
| BAC24 | NA03_Bacteria_65_16 | Bacteria | 3,910,000 | 65.04% | 63 | 3719 | 91.45% |
| BAC25 | anamox1_Bacteria_50_18_curated | Bacteria | 820,570 | 49.55% | 22 | 874 | 63.77% |
| BAC26 | anamox1_Bacteria_72_15_curated | Bacteria | 2,940,000 | 72.45% | 24 | 2507 | 93.75% |
| BAC27 | anamox1_Bacteria_53_17_curated | Bacteria | 3,470,000 | 53.24% | 12 | 2895 | 95.91% |
| BAC28 | anamox1_Bacteria_57_32_curated | Bacteria | 2,940,000 | 56.91% | 76 | 2571 | 92.02% |
| BAC29 | anamox3_Bacteria_67_13_curated | Bacteria | 1,640,000 | 66.50% | 47 | 1442 | 92.02% |
| BACG11 | anamox1_Sphingobacteriales_43_8_curated | Bacteroidetes | 3,160,000 | 42.95% | 130 | 2826 | 95.13% |
| BACT01 | LAC_NA08_Flavobacteriales_41_5 | Bacteroidetes | 1,240,000 | 41.09% | 665 | 1648 | 50.87% |
| BACT02 | LAC_NA11_Bacteroidales_40_6 | Bacteroidetes | 2,280,000 | 40.14% | 900 | 2540 | 70.85% |
| BACT03 | NA04_Bacteroidetes_30_9 | Bacteroidetes | 2,580,000 | 30.05% | 248 | 2356 | 95.63% |
| BACT04 | LAC_NA06_Sphingobacteriales_42_58 | Bacteroidetes | 3,450,000 | 42.01% | 34 | 2968 | 97.04% |
| BACT05 | NA04_Sphingobacteriales_44_8 | Bacteroidetes | 4,070,000 | 43.73% | 474 | 3624 | 86.45% |
| BACT06 | NA04_BJP_IG2103_Bacteroidetes_37_22_46_30 | Bacteroidetes | 2,930,000 | 45.76% | 35 | 2441 | 96.77% |
| BACT07 | anamox1_Bacteroidetes_63_11_curated | Bacteroidetes | 3,360,000 | 63.21% | 73 | 2915 | 99.46% |
| BACT08 | anamox2_Sphingobacteriales_41_11_curated | Bacteroidetes | 2,770,000 | 41.16% | 129 | 2490 | 85.67% |
| BACT09 | anamox4_Bacteroidetes_40_74_curated | Bacteroidetes | 2,630,000 | 39.91% | 15 | 2219 | 99.52% |
| BACT10 | anamox3_Sphingobacteriales_50_9_curated | Bacteroidetes | 4,310,000 | 49.73% | 216 | 2999 | 95.45% |
| BACT12 | anamox3_Bacteroidetes_39_15_curated | Bacteroidetes | 2,530,000 | 39.26% | 23 | 2188 | 98.57% |
| BACT13 | anamox1_Bacteroidetes_39_16_curated | Bacteroidetes | 2,900,000 | 38.84% | 40 | 2472 | 98.73% |
| BAC17 | anamox1_Bacteria_55_18_curated | Bacteria | 4,100,000 | 54.56% | 216 | 3365 | 99.46% |
| CFLX01 | LAC_NA10_Chloroflexi_62_239 | Chloroflexi | 1,430,000 | 61.97% | 417 | 1859 | 49.63% |
| CFLX02 | LAC_NA08_Chloroflexi_60_15 | Chloroflexi | 3,260,000 | 59.74% | 310 | 3038 | 86.36% |
| CFLX03 | NA01_Chloroflexi_65_16 | Chloroflexi | 3,700,000 | 65.08% | 207 | 3252 | 93.64% |
| CFLX04 | NA03_Anaerolineales_41_23 | Chloroflexi | 2,360,000 | 41.48% | 215 | 2322 | 85.45% |
| CFLX05 | LAC_NA08_Chloroflexi_58_8 | Chloroflexi | 2,650,000 | 57.66^ | 432 | 2733 | 74.18% |
| CFLX06 | LAC_NA08_Chloroflexi_67_31 | Chloroflexi | 3,550,000 | 66.65% | 70 | 2905 | 90.91% |
| CFLX07 | anamox3_Chloroflexi_59_6_curated | Chloroflexi | 1,410,000 | 53.68% | 351 | 1507 | 32.37% |
| CFLX08 | NA03_RBG_16_Chloroflexi_57_11_curated_55_21 | Chloroflexi | 3,440,000 | 55.06% | 367 | 3313 | 91.2% |
| CFLX09 | LAC_NA07_Chloroflexi_57_9 | Chloroflexi | 2,090,000 | 56.86% | 325 | 2202 | 70.11% |
| CFLX10 | LAC_NA11_Caldilinea_aerophila_60_6 | Chloroflexi | 3,520,000 | 59.88% | 1263 | 3974 | 70.72% |
| CFLX11 | LAC_NA07_Chloroflexi_66_23 | Chloroflexi | 4,470,000 | 66.09% | 141 | 3687 | 94.55% |
| CFLX12 | NA01_Caldilinea_aerophila_61_10 | Chloroflexi | 3,730,000 | 60.76% | 159 | 2098 | 83.64% |
| CFLX13 | NA02_Anaerolinea_thermophila_56_16 | Chloroflexi | 2,140,000 | 56.04% | 540 | 2398 | 78.33% |
| CFLX14 | anamox3_Chloroflexi_68_6_curated | Chloroflexi | 2,290,000 | 67.35% | 479 | 2330 | 56.04% |
| CFLX15 | LAC_NA07_RBG_16_RIF_CHLX_72_14_curated_75_20 | Chloroflexi | 2,490,000 | 74.73% | 63 | 2327 | 91.20% |
| CFLX16 | LAC_NA07_Chloroflexi_60_59 | Chloroflexi | 3,860,000 | 60.11% | 13 | 3453 | 89.55% |
| CFLX17 | anamox1_Chloroflexi_52_59_curated | Chloroflexi | 2,860,000 | 52.47% | 96 | 2759 | 92.73% |
| CFLX18 | anamox2_Chloroflexi_60_8_curated | Chloroflexi | 2,020,000 | 60.06% | 111 | 1885 | 44.42% |
| CLA01 | anamox1_Candidatus_Cloacimonas_acidaminovorans_38_6_curated | Candidatus Cloacimonas | 1,190,000 | 35.28% | 246 | 1132 | 53.43% |
| CPR01 | LAC_NA11_Microgenomates_50_64 | Microgenomates | 869,170 | 49.81% | 63 | 986 | 55.88% |
| CPR02 | anamox2_Microgenomates_45_6_curated | Microgenomates | 740,220 | 44.28% | 99 | 904 | 59.54% |
| CPR03 | NA01_Roizmanbacteria_38_28 | Microgenomates | 897,990 | 38.12% | 17 | 921 | 68.93% |
| CPR04 | NA04_Candidatus_Saccharibacteria_41_123 | Microgenomates | 820,330 | 40.66% | 14 | 857 | 66.82% |
| CPR05 | anamox4_Microgenomates_48_8_curated | Microgenomates | 999,880 | 47.64% | 49 | 1055 | 63.3% |
| CPR06 | LAC_NA07_Roizmannbacteria_52_60 | Microgenomates | 885,170 | 52.20% | 1 | 945 | 73.98% |
| CPR07 | LAC_NA06_Microgenomates_41_17 | Microgenomates | 1,100,000 | 41.16% | 6 | 1203 | 67.89% |
| DADA01 | LAC_NA06_RIFCSPHIGHO2_12_FULL_Dadabacteria_53_21_curated_58_6 | Dadabacteria | 918,620 | 57.55% | 539 | 1314 | 43.08% |
| DEN01 | LAC_NA07_Truepera_radiovictrix_72_29 | Deinococcus-Thermus | 1,320,000 | 72.29% | 165 | 1356 | 73.23% |
| GEM01 | anamox1_Gemmatimonas_aurantiaca_57_6_curated | Gemmatimonadota | 2,630,000 | 56.94% | 255 | 2549 | 90.66% |
| IGN01 | LAC_NA08_Ignavibacterium_album_33_266 | Ignavibacteriae | 2,140,000 | 33.41% | 731 | 2621 | 67.42% |
| IGN02 | anamox3_sub_Ignavibacteriales_42_14_curated | Ignavibacteriae | 3,160,000 | 42.21% | 22 | 2498 | 95.63% |
| IGN03 | anamox2_Ignavibacteriales_33_9_curated | Ignavibacteriae | 2,940,000 | 33.09% | 166 | 2753 | 76.8% |
| IGN04 | LAC_NA11_Ignavibacteriales_56_75 | Ignavibacteriae | 2,990,000 | 56.40% | 21 | 2685 | 96.72% |
| IGN05 | NA03_BJP_IG2069_Ignavibacteriae_38_11_31_13 | Ignavibacteriae | 2,740,000 | 30.53% | 53 | 2344 | 96.89% |
| IGN06 | anamox2_Ignavibacteriales_41_12_curated | Ignavibacteriae | 3,160,000 | 41.32% | 42 | 2805 | 95.63% |
| IGN07 | anamox1_RBG_16_Ignavibacteria_36_9_curated_35_5_curated | Ignavibacteriae | 1,160,000 | 34.03% | 347 | 1374 | 51.34% |
| MYX01 | NA04_Myxococcales_72_13 | Myxococcota | 4,300,000 | 71.89% | 121 | 3695 | 89.03# |
| MYX02 | anamox2_Myxococcales_71_5_curated | Myxococcota | 1,430,000 | 70.21% | 495 | 1687 | 24.99% |
| NSP01 | anamox4_Candidatus_Nitrospira_defluvii_60_9_curated | Nitrospirota | 3,090,000 | 60.30% | 94 | 3084 | 95.85% |
| PROT01 | NA04_Burkholderiales_70_137 | Proteobacteria | 2,510,000 | 69.69% | 131 | 2498 | 90.41% |
| PROT02 | NA02_Rhizobiales_66_27 | Proteobacteria | 2,950,000 | 66.38% | 163 | 3000 | 95.3% |
| PROT03 | NA04_Rhodocyclales_67_27 | Proteobacteria | 2,480,000 | 66.98% | 295 | 2718 | 85.24% |
| PROT04 | LAC_NA06_Betaproteobacteria_71_7 | Proteobacteria | 4,100,000 | 70.65% | 1841 | 5661 | 97.98% |
| PROT05 | LAC_NA07_Rhodobacterales_68_7 | Proteobacteria | 1,330,000 | 68.41% | 473 | 1674 | 58.41% |
| PROT06 | NA01_Rhizobiales_69_9 | Proteobacteria | 3,340,000 | 69.28% | 447 | 3602 | 79.77% |
| PROT07 | LAC_NA10_Xanthomonadales_68_7 | Proteobacteria | 2,460,000 | 68.28% | 555 | 2584 | 68.74% |
| PROT08 | LAC_NA10_Lysobacter_70_8 | Proteobacteria | 1,030,000 | 70.00% | 294 | 1180 | 37.95% |
| PROT09 | LAC_NA06_Xanthomonadales_70_10 | Proteobacteria | 1,920,000 | 69.75% | 377 | 2039 | 69.12% |
| PROT10 | anamox1_Betaproteobacteria_69_7_curated | Proteobacteria | 2,110,000 | 66.39% | 531 | 2636 | 52.65% |
| PROT11 | anamox2_Burkholderiales_67_5_curated | Proteobacteria | 1,250,000 | 66.28% | 298 | 1434 | 38.05% |
| PROT12 | LAC_NA08_Gammaproteobacteria_68_22 | Proteobacteria | 2,800,000 | 68.43% | 13 | 2594 | 89.07% |
| PROT13 | NA03_Alphaproteobacteria_58_599 | Proteobacteria | 2,550,000 | 57.83% | 1 | 2485 | 95.81% |
| PROT14 | LAC_NA09_Gammaproteobacteria_70_24 | Proteobacteria | 2,570,000 | 69.91% | 23 | 2420 | 88.23% |
| PROT15 | LAC_NA11_Betaproteobacteria_62_13 | Proteobacteria | 2,820,000 | 62.38% | 76 | 2811 | 97.47% |
| PROT16 | anamox1_Burkholderiales_71_17_curated | Proteobacteria | 3,640,000 | 70.81% | 114 | 3448 | 61.64% |
| PROT17 | LAC_NA06_Burkholderiales_73_13 | Proteobacteria | 3,680,000 | 72.87% | 335 | 3453 | 89.44% |
| PROT18 | anamox4_Gammaproteobacteria_67_14_curated | Proteobacteria | 3,270,000 | 66.91% | 73 | 3049 | 92.53% |
| PROT19 | anamox2_Burkholderiales_68_9_curated | Proteobacteria | 3,530,000 | 68.25% | 181 | 3452 | 95.74% |
| PROT20 | anamox3_Nitrosomonas_europaea_51_28_curated | Proteobacteria | 2,370,000 | 50.54% | 93 | 2302 | 95.55% |
| PROT21 | NA03_Alphaproteobacteria_61_24 | Proteobacteria | 3,390,000 | 61.02% | 12 | 3189 | 99.50% |
| PROT23 | anamox2_Burkholderiales_70_13_curated | Proteobacteria | 3,070,000 | 70.33% | 39 | 2841 | 87.2% |
| PROT24 | anamox1_Proteobacteria_67_8_curated | Proteobacteria | 2,910,000 | 66.93% | 203 | 2910 | 83.43% |
| PROT25 | anamox2_Xanthomonadales_68_9_curated | Proteobacteria | 1,240,000 | 67.63% | 21 | 1168 | 55.44% |
| PROT26 | anamox2_Rhizobiales_67_45_curated | Proteobacteria | 4,840,000 | 66.56% | 24 | 4668 | 98.36% |
| PROT27 | anamox1_Nitrosomonas_europaea_50_14_curated | Proteobacteria | 2,130,000 | 50.43% | 37 | 2006 | 99.74% |
| PROT28 | anamox2_Hydrogenophilales_66_19_curated | Proteobacteria | 2,260,000 | 65.94% | 122 | 2331 | 87.57% |
| SPR01 | NA03_Leptonema_illini_45_7 | Spirochaetes | 2,310,000 | 45.31% | 383 | 2382 | 77.25% |
| SPR02 | NA04_Turneriella_parva_44_15 | Spirochaetes | 2,930,000 | 44.39% | 65 | 2790 | 93.19% |
| VER01 | anamox2_Verrucomicrobia_62_8_curated | Verrucomicrobia | 3,690,000 | 61.78% | 161 | 3134 | 95.27% |
| VER02 | anamox3_Pedosphaera_parvula_66_5_curated | Verrucomicrobia | 1,230,000 | 65.98% | 543 | 1384 | 52.71% |
| VER03 | anamox2_Verrucomicrobia_58_8_curated | Verrucomicrobia | 3,590,000 | 57.68% | 250 | 3293 | 89.46% |
| VER04 | anamox3_Verrucomicrobia_59_12_curated | Verrucomicrobia | 2,710,000 | 59.27% | 50 | 2592 | 96.62% |

SI Table 6: MAG D37/D232 LR Changes

| MAG | RFG1 | RFG2 | RFG3 |
| --- | --- | --- | --- |
| anamox1_Acidobacteria_curated | -1.77982 | -1.3459 | -1.03193 |
| anamox1__Bacteria_33_9_curated | 0 | 0.433921 | 0.747891 |
| anamox1_Bacteria_45_8_curated | 1.264691 | 1.698612 | 2.012582 |
| anamox1_Bacteria_50_18_curated | 0.064567 | 0.498488 | 0.812458 |
| anamox1_Bacteria_53_17_curated | -2.27411 | -1.84019 | -1.52622 |
| anamox1_Bacteria_55_18_curated | -2.58838 | -2.15446 | -1.84049 |
| anamox1_Bacteria_56_37_curated | -0.63401 | -0.20009 | 0.113882 |
| anamox1_Bacteria_57_32_curated | -3.22817 | -2.79425 | -2.48028 |
| anamox1_Bacteria_57_9_curated | -1.2124 | -0.77848 | -0.46451 |
| anamox1_Bacteria_64_7_curated | -1.40103 | -0.96711 | -0.65314 |
| anamox1_Bacteria_65_5_curated | -0.03475 | 0.399174 | 0.713143 |
| anamox1_Bacteria_72_15_curated | -2.77627 | -2.34235 | -2.02838 |
| anamox1_Bacteroidetes_39_16_curated | -3.10719 | -2.67327 | -2.3593 |
| anamox1_Bacteroidetes_63_11_curated | 1.197916 | 1.631837 | 1.945806 |
| anamox1_Betaproteobacteria_69_7_curated | -1.80718 | -1.37325 | -1.05928 |
| anamox1_Burkholderiales_71_17_curated | -0.58284 | -0.14892 | 0.165054 |
| anamox1_Candidatus_Cloacimonas_acidaminovorans_38_6_curated | 1.042035 | 1.475956 | 1.789925 |
| anamox1_Chloroflexi_52_59_curated | -0.43994 | -0.00602 | 0.307946 |
| anamox1_Gemmatimonas_aurantiaca_57_6_curated | -0.13016 | 0.303763 | 0.617732 |
| anamox1_Nitrosomonas_europaea_50_14_curated | -1.77595 | -1.34203 | -1.02806 |
| anamox1_Proteobacteria_67_8_curated | -1.87713 | -1.44321 | -1.12924 |
| anamox1_RBG_16_Ignavibacteria_36_9_curated_35_5_curated | 0.187597 | 0.621518 | 0.935488 |
| anamox1_Sphingobacteriales_43_8_curated | -2.65684 | -2.22292 | -1.90895 |
| anamox2_Actinobacteria_65_5_curated | -0.83256 | -0.39864 | -0.08467 |
| anamox2_Bacteria_61_6_curated | 1.299484 | 1.733405 | 2.047375 |
| anamox2_Bacteria_68_6_curated | 0.221993 | 0.655914 | 0.969884 |
| anamox2_Burkholderiales_67_5_curated | 0.930401 | 1.364322 | 1.678291 |
| anamox2_Burkholderiales_68_9_curated | -0.73692 | -0.303 | 0.010969 |
| anamox2_Burkholderiales_70_13_curated | 0.501921 | 0.935842 | 1.249812 |
| anamox2_Chloroflexi_60_8_curated | -1.48376 | -1.04984 | -0.73587 |
| anamox2_Hydrogenophilales_66_19_curated | 1.051323 | 1.485244 | 1.799213 |
| anamox2_Ignavibacteriales_33_9_curated | 0.405233 | 0.839154 | 1.153123 |
| anamox2_Ignavibacteriales_41_12_curated | 0.216878 | 0.650799 | 0.964768 |
| anamox2_Methanosarcina_thermophila_41_9_curated | -2.18282 | -1.7489 | -1.43493 |
| anamox2_Microgenomates_45_6_curated | -0.70318 | -0.26926 | 0.044711 |
| anamox2_Myxococcales_71_5_curated | 0.623163 | 1.057084 | 1.371054 |
| anamox2_Rhizobiales_67_45_curated | 0.911623 | 1.345544 | 1.659514 |
| anamox2_Sphingobacteriales_41_11_curated | 0.042353 | 0.476274 | 0.790243 |
| anamox2_Verrucomicrobia_58_8_curated | -0.26585 | 0.168076 | 0.482045 |
| anamox2_Verrucomicrobia_62_8_curated | -0.43392 | 0 | 0.313969 |
| anamox2_Xanthomonadales_68_9_curated | -0.19986 | 0.234057 | 0.548027 |
| anamox3_Acidobacteria_71_4_curated | -1.46911 | -1.03519 | -0.72122 |
| anamox3_Bacteria_66_7_curated | -0.10137 | 0.332552 | 0.646521 |
| anamox3_Bacteria_67_13_curated | -0.90291 | -0.46899 | -0.15502 |
| anamox3_Bacteria_67_15_curated | -1.4235 | -0.98958 | -0.67561 |
| anamox3_Bacteroidetes_39_15_curated | 0.403564 | 0.837485 | 1.151454 |
| anamox3_Chloroflexi_59_6_curated | -0.90286 | -0.46894 | -0.15497 |
| anamox3_Chloroflexi_68_6_curated | -0.71449 | -0.28057 | 0.033398 |
| anamox3_Nitrosomonas_europaea_51_28_curated | -2.81727 | -2.38335 | -2.06938 |
| anamox3_Pedosphaera_parvula_66_5_curated | -1.46866 | -1.03474 | -0.72077 |
| anamox3_Sphingobacteriales_50_9_curated | -2.78219 | -2.34827 | -2.0343 |
| anamox3_sub_Ignavibacteriales_42_14_curated | 1.450668 | 1.884589 | 2.198558 |
| anamox3_Verrucomicrobia_59_12_curated | -4.41896 | -3.98504 | -3.67107 |
| anamox4_Bacteria_63_7_curated | -3.09306 | -2.65913 | -2.34516 |
| anamox4_Bacteroidetes_40_74_curated | -3.56254 | -3.12861 | -2.81465 |
| anamox4_Candidatus_Nitrospira_defluvii_60_9_curated | -1.34792 | -0.914 | -0.60003 |
| anamox4_Gammaproteobacteria_67_14_curated | -3.16664 | -2.73271 | -2.41874 |
| anamox4_Microgenomates_48_8_curated | -2.90478 | -2.47086 | -2.15689 |
| anamox4_sub_Candidatus_Brocadia_sinica_42_75_curated | -0.43375 | 0.000174 | 0.314144 |
| LAC_NA06_Acidobacteria_54_19 | -2.20477 | -1.77085 | -1.45688 |
| LAC_NA06_Actinobacteria_71_15 | -0.4978 | -0.06388 | 0.250086 |
| LAC_NA06_Betaproteobacteria_71_7 | -0.90021 | -0.46629 | -0.15232 |
| LAC_NA06_Burkholderiales_73_13 | -1.58571 | -1.15179 | -0.83782 |
| LAC_NA06_Candidatus_Solibacter_usitatus_62_12 | -0.8555 | -0.42158 | -0.10761 |
| LAC_NA06_Microgenomates_41_17 | -2.53461 | -2.10069 | -1.78672 |
| LAC_NA06_RIFCSPHIGHO2_12_FULL_Dadabacteria_53_21_curated_58_6 | -1.45 | -1.01608 | -0.70211 |
| LAC_NA06_Sphingobacteriales_42_58 | -1.08037 | -0.64644 | -0.33247 |
| LAC_NA06_Xanthomonadales_70_10 | -0.24986 | 0.184064 | 0.498034 |
| LAC_NA07_Actinobacteria_74_18 | -1.19828 | -0.76436 | -0.45039 |
| LAC_NA07_Actinotalea_fermentans_75_19 | -2.31665 | -1.88273 | -1.56876 |
| LAC_NA07_Bacteria_38_171 | -1.59721 | -1.16329 | -0.84932 |
| LAC_NA07_Bacteria_57_12 | -1.45178 | -1.01786 | -0.70389 |
| LAC_NA07_Bacteria_70_305 | -0.24661 | 0.187308 | 0.501277 |
| LAC_NA07_Bacteria_71_12 | -2.55738 | -2.12345 | -1.80948 |
| LAC_NA07_Chloroflexi_57_9 | -0.01693 | 0.416995 | 0.730965 |
| LAC_NA07_Chloroflexi_60_59 | -0.70041 | -0.26649 | 0.047483 |
| LAC_NA07_Chloroflexi_66_23 | -1.81055 | -1.37663 | -1.06266 |
| LAC_NA07_RBG_16_RIF_CHLX_72_14_curated_75_20 | -2.16044 | -1.72652 | -1.41255 |
| LAC_NA07_Rhodobacterales_68_7 | -0.1535 | 0.280423 | 0.594393 |
| LAC_NA07_Roizmannbacteria_52_60 | -2.1934 | -1.75948 | -1.44551 |
| LAC_NA07_Truepera_radiovictrix_72_29 | -1.28346 | -0.84954 | -0.53557 |
| LAC_NA08_Chloroflexi_58_8 | -0.58222 | -0.1483 | 0.16567 |
| LAC_NA08_Chloroflexi_60_15 | -1.8236 | -1.38968 | -1.07571 |
| LAC_NA08_Chloroflexi_67_31 | 0.060378 | 0.494299 | 0.808268 |
| LAC_NA08_Flavobacteriales_41_5 | 1.271384 | 1.705305 | 2.019275 |
| LAC_NA08_Gammaproteobacteria_68_22 | -0.74789 | -0.31397 | 0 |
| LAC_NA08_Ignavibacterium_album_33_266 | 0.273581 | 0.707502 | 1.021472 |
| LAC_NA09_Acidobacteria_70_95 | 0.51073 | 0.944651 | 1.25862 |
| LAC_NA09_Gammaproteobacteria_70_24 | 4.50999 | 4.943911 | 5.257881 |
| LAC_NA10_Actinobacteria_74_12 | 1.269679 | 1.7036 | 2.01757 |
| LAC_NA10_Bacteria_62_9 | 3.396389 | 3.83031 | 4.14428 |
| LAC_NA10_Chloroflexi_62_239 | 0.348056 | 0.781977 | 1.095946 |
| LAC_NA10_Lysobacter_70_8 | 1.889056 | 2.322977 | 2.636946 |
| LAC_NA10_Xanthomonadales_68_7 | 1.836755 | 2.270676 | 2.584646 |
| LAC_NA11_Bacteria_34_14 | 3.864204 | 4.298125 | 4.612094 |
| LAC_NA11_Bacteria_57_35 | 1.070316 | 1.504237 | 1.818206 |
| LAC_NA11_Bacteroidales_40_6 | 1.204191 | 1.638112 | 1.952081 |
| LAC_NA11_Betaproteobacteria_62_13 | 4.629397 | 5.063318 | 5.377288 |
| LAC_NA11_Caldilinea_aerophila_60_6 | 1.559157 | 1.993078 | 2.307048 |
| LAC_NA11_Ignavibacteriales_56_75 | 2.816129 | 3.25005 | 3.56402 |
| LAC_NA11_Microgenomates_50_64 | 2.29927 | 2.733191 | 3.04716 |
| NA01_Acidobacteria_59_12 | 0.693025 | 1.126946 | 1.440915 |
| NA01_Bacteria_60_23 | 1.999411 | 2.433332 | 2.747302 |
| NA01_Caldilinea_aerophila_61_10 | -0.36419 | 0.069729 | 0.383698 |
| NA01_Chloroflexi_65_16 | -1.47319 | -1.03927 | -0.7253 |
| NA01_Fimbriimonas_ginsengisoli_61_16 | -0.01282 | 0.421102 | 0.735072 |
| NA01_Rhizobiales_69_9 | 1.44392 | 1.877841 | 2.19181 |
| NA01_Roizmanbacteria_38_28 | 1.515339 | 1.94926 | 2.26323 |
| NA02_Anaerolinea_thermophila_56_16 | 3.002677 | 3.436598 | 3.750568 |
| NA02_Rhizobiales_66_27 | 0.100987 | 0.534908 | 0.848877 |
| NA03_Alphaproteobacteria_58_599 | -0.88757 | -0.45364 | -0.13967 |
| NA03_Alphaproteobacteria_61_24 | 3.589824 | 4.023746 | 4.337715 |
| NA03_Anaerolineales_41_23 | 0.88398 | 1.317901 | 1.631871 |
| NA03_Bacteria_65_16 | 0.833982 | 1.267903 | 1.581873 |
| NA03_BJP_IG2069_Ignavibacteriae_38_11_31_13 | -1.2438 | -0.80988 | -0.49591 |
| NA03_Leptonema_illini_45_7 | -0.33367 | 0.100248 | 0.414218 |
| NA03_RBG_16_Chloroflexi_57_11_curated_55_21 | 1.781768 | 2.215689 | 2.529658 |
| NA04_Bacteria_50_51 | 1.860578 | 2.294499 | 2.608469 |
| NA04_Bacteria_58_19 | -0.11038 | 0.323543 | 0.637513 |
| NA04_Bacteria_65_10 | 1.142646 | 1.576567 | 1.890536 |
| NA04_Bacteria_69_30 | 0.440185 | 0.874106 | 1.188076 |
| NA04_Bacteroidetes_30_9 | 2.540491 | 2.974412 | 3.288382 |
| NA04_BJP_IG2103_Bacteroidetes_37_22_46_30 | 3.224898 | 3.658819 | 3.972789 |
| NA04_Burkholderiales_70_137 | 0.124463 | 0.558384 | 0.872353 |
| NA04_Candidatus_Saccharibacteria_41_123 | 2.871607 | 3.305528 | 3.619498 |
| NA04_Myxococcales_72_13 | 3.460994 | 3.894915 | 4.208885 |
| NA04_Rhodocyclales_67_27 | 1.250777 | 1.684698 | 1.998667 |
| NA04_Sphingobacteriales_44_8 | 2.136662 | 2.570583 | 2.884552 |
| NA04_Turneriella_parva_44_15 | 2.238999 | 2.67292 | 2.98689 |

|  | Upper Limit | Lower Limit |
| --- | --- | --- |
| RFG1 (anamox1_Bacteria_33_9_curated) | 0.143726 | -0.47735 |
| RFG2 (anamox2_Verrucomicrobia_62_8_curated) | 0.577647 | -0.04343 |
| RFG3 (LAC_NA08_Gammaproteobacteria_68_22) | 0.891617 | 0.270544 |

|  |  |  |
| --- | --- | --- |
|  | SI Table 7: Log ratio changes confidence intervals |  |
|  |  |  |
|  |  |  |

SI Table 8: MAG RPKM Values

| MAG | D37 | D140 | D232 | totals |
| --- | --- | --- | --- | --- |
| anamox1_Acidobacteria_62_5_curated | 0.006211 | 0.002572 | 0.001277 | 0.01006 |
| anamox1_Bacteria_33_9_curated | 0.003004 | 0.003106 | 0.00369 | 0.0098 |
| anamox1_Bacteria_45_8_curated | 9.90E-05 | 0.000176 | 0.00045 | 0.000725 |
| anamox1_Bacteria_50_18_curated | 0.000115 | 3.91E-05 | 0.000151 | 0.000306 |
| anamox1_Bacteria_53_17_curated | 8.81E-05 | 5.15E-05 | 1.15E-05 | 0.000151 |
| anamox1_Bacteria_55_18_curated | 0.001926 | 0.001072 | 0.000186 | 0.003184 |
| anamox1_Bacteria_56_37_curated | 0.528339 | 0.288761 | 0.34461 | 1.16171 |
| anamox1_Bacteria_57_32_curated | 2.08E-05 | 1.03E-05 | 2.93E-06 | 3.40E-05 |
| anamox1_Bacteria_57_9_curated | 0.000327 | 0.00027 | 0.00012 | 0.000717 |
| anamox1_Bacteria_64_7_curated | 0.001251 | 0.000816 | 0.000401 | 0.002469 |
| anamox1_Bacteria_65_5_curated | 0.000789 | 0.000762 | 0.000878 | 0.002429 |
| anamox1_Bacteria_72_15_curated | 0.000124 | 2.36E-05 | 1.33E-05 | 0.000161 |
| anamox1_Bacteroidetes_39_16_curated | 8.44E-06 | 2.51E-06 | 8.57E-07 | 1.18E-05 |
| anamox1_Bacteroidetes_63_11_curated | 0.002264 | 0.008341 | 0.009036 | 0.019642 |
| anamox1_Betaproteobacteria_69_7_curated | 0.111081 | 0.026609 | 0.022672 | 0.160362 |
| anamox1_Burkholderiales_71_17_curated | 0.007112 | 0.014539 | 0.004898 | 0.02655 |
| anamox1_Candidatus_Cloacimonas_acidaminovorans_38_6_curated | 2.38E-06 | 3.05E-06 | 6.20E-06 | 1.16E-05 |
| anamox1_Chloroflexi_52_59_curated | 0.030156 | 0.025691 | 0.024239 | 0.080086 |
| anamox1_Gemmatimonas_aurantiaca_57_6_curated | 0.003277 | 0.015437 | 0.003565 | 0.02228 |
| anamox1_Nitrosomonas_europaea_50_14_curated | 0.000246 | 0.000106 | 5.48E-05 | 0.000407 |
| anamox1_Proteobacteria_67_8_curated | 0.001968 | 0.000821 | 0.00038 | 0.003169 |
| anamox1_RBG_16_Ignavibacteria_36_9_curated_35_5_curated | 0.000651 | 0.000523 | 0.000942 | 0.002116 |
| anamox1_Sphingobacteriales_43_8_curated | 0.001037 | 0.001041 | 9.11E-05 | 0.002169 |
| anamox2_Actinobacteria_65_5_curated | 0.07197 | 0.052252 | 0.038705 | 0.162927 |
| anamox2_Bacteria_61_6_curated | 0.000155 | 0.000238 | 0.000674 | 0.001067 |
| anamox2_Bacteria_68_6_curated | 0.045385 | 0.204178 | 0.069537 | 0.3191 |
| anamox2_Burkholderiales_67_5_curated | 0.013811 | 0.018652 | 0.042328 | 0.074791 |
| anamox2_Burkholderiales_68_9_curated | 0.005229 | 0.011966 | 0.003099 | 0.020294 |
| anamox2_Burkholderiales_70_13_curated | 0.000834 | 0.00113 | 0.001606 | 0.00357 |
| anamox2_Chloroflexi_60_8_curated | 0.003463 | 0.001735 | 0.000965 | 0.006163 |
| anamox2_Hydrogenophilales_66_19_curated | 0.000727 | 0.001098 | 0.002463 | 0.004288 |
| anamox2_Ignavibacteriales_33_9_curated | 0.108968 | 0.150627 | 0.202631 | 0.462227 |
| anamox2_Ignavibacteriales_41_12_curated | 0.000603 | 0.001402 | 0.000918 | 0.002922 |
| anamox2_Methanosarcina_thermophila_41_9_curated | 8.54E-05 | 4.04E-05 | 1.38E-05 | 0.00014 |
| anamox2_Microgenomates_45_6_curated | 0.163884 | 0.078205 | 0.100563 | 0.342652 |
| anamox2_Myxococcales_71_5_curated | 0.000936 | 0.002248 | 0.00198 | 0.005164 |
| anamox2_Rhizobiales_67_45_curated | 0.000114 | 0.000105 | 0.000326 | 0.000545 |
| anamox2_Sphingobacteriales_41_11_curated | 0.002197 | 0.002492 | 0.002818 | 0.007507 |
| anamox2_Verrucomicrobia_58_8_curated | 0.000181 | 0.000218 | 0.000165 | 0.000564 |
| anamox2_Verrucomicrobia_62_8_curated | 0.007203 | 0.006882 | 0.005759 | 0.019844 |
| anamox2_Xanthomonadales_68_9_curated | 0.00024 | 0.000912 | 0.000247 | 0.001398 |
| anamox3_Acidobacteria_71_4_curated | 0.023815 | 0.012341 | 0.006766 | 0.042921 |
| anamox3_Bacteria_66_7_curated | 0.000631 | 0.001628 | 0.000695 | 0.002954 |
| anamox3_Bacteria_67_13_curated | 3.35E-06 | 3.14E-06 | 2.36E-06 | 8.85E-06 |
| anamox3_Bacteria_67_15_curated | 0.00892 | 0.002728 | 0.002638 | 0.014286 |
| anamox3_Bacteroidetes_39_15_curated | 2.22E-05 | 2.86E-05 | 3.50E-05 | 8.58E-05 |
| anamox3_Chloroflexi_59_6_curated | 0.351935 | 0.190165 | 0.175534 | 0.717635 |
| anamox3_Chloroflexi_68_6_curated | 0.061493 | 0.034993 | 0.037099 | 0.133585 |
| anamox3_Nitrosomonas_europaea_51_28_curated | 0.006325 | 0.00274 | 0.000472 | 0.009537 |
| anamox3_Pedosphaera_parvula_66_5_curated | 0.001103 | 0.000457 | 0.000332 | 0.001892 |
| anamox3_Sphingobacteriales_50_9_curated | 0.001807 | 0.001235 | 0.000151 | 0.003194 |
| anamox3_sub_Ignavibacteriales_42_14_curated | 0.092131 | 0.326489 | 0.491841 | 0.91046 |
| anamox3_Verrucomicrobia_59_12_curated | 2.75E-05 | 4.70E-06 | 2.02E-06 | 3.42E-05 |
| anamox4_Bacteria_63_7_curated | 0.233667 | 0.047579 | 0.012995 | 0.294241 |
| anamox4_Bacteroidetes_40_74_curated | 0.002813 | 0.000743 | 9.98E-05 | 0.003655 |
| anamox4_Candidatus_Nitrospira_defluvii_60_9_curated | 0.001149 | 0.00072 | 0.000367 | 0.002236 |
| anamox4_Gammaproteobacteria_67_14_curated | 0.017333 | 0.003895 | 0.0009 | 0.022128 |
| anamox4_Microgenomates_48_8_curated | 0.004511 | 0.000962 | 0.000319 | 0.005793 |
| anamox4_sub_Candidatus_Brocadia_sinica_42_75_curated | 9.672443 | 10.25377 | 7.805721 | 27.73194 |
| LAC_NA06_Acidobacteria_54_19 | 0.003664 | 0.001198 | 0.000497 | 0.005358 |
| LAC_NA06_Actinobacteria_71_15 | 0.059425 | 0.027907 | 0.044429 | 0.131762 |
| LAC_NA06_Betaproteobacteria_71_7 | 0.423245 | 0.279806 | 0.212422 | 0.915474 |
| LAC_NA06_Burkholderiales_73_13 | 0.015869 | 0.005565 | 0.00419 | 0.025624 |
| LAC_NA06_Candidatus_Solibacter_usitatus_62_12 | 0.099705 | 0.07025 | 0.051995 | 0.221949 |
| LAC_NA06_Microgenomates_41_17 | 0.001591 | 0.001032 | 0.000158 | 0.00278 |
| LAC_NA06_RIFCSPHIGHO2_12_FULL_Dadabacteria_53_21_curated_58_6 | 0.039493 | 0.009404 | 0.011482 | 0.060379 |
| LAC_NA06_Sphingobacteriales_42_58 | 0.037026 | 0.013769 | 0.015586 | 0.066381 |
| LAC_NA06_Xanthomonadales_70_10 | 0.040064 | 0.11222 | 0.038219 | 0.190503 |
| LAC_NA07_Actinobacteria_74_18 | 0.162363 | 0.058828 | 0.061087 | 0.282277 |
| LAC_NA07_Actinotalea_fermentans_75_19 | 0.041313 | 0.017182 | 0.005042 | 0.063537 |
| LAC_NA07_Bacteria_38_171 | 0.287491 | 0.067699 | 0.072364 | 0.427554 |
| LAC_NA07_Bacteria_57_12 | 0.221275 | 0.106413 | 0.063713 | 0.391402 |
| LAC_NA07_Bacteria_70_305 | 1.186839 | 1.698255 | 1.139331 | 4.024425 |
| LAC_NA07_Bacteria_71_12 | 0.167489 | 0.035013 | 0.015919 | 0.218421 |
| LAC_NA07_Chloroflexi_57_9 | 0.20102 | 0.154175 | 0.243924 | 0.599118 |
| LAC_NA07_Chloroflexi_60_59 | 0.056022 | 0.00375 | 0.034162 | 0.093933 |
| LAC_NA07_Chloroflexi_66_23 | 0.227458 | 0.069382 | 0.045569 | 0.342409 |
| LAC_NA07_RBG_16_RIF_CHLX_72_14_curated_75_20 | 0.086919 | 0.019389 | 0.012342 | 0.118651 |
| LAC_NA07_Rhodobacterales_68_7 | 0.216367 | 0.194236 | 0.229316 | 0.639918 |
| LAC_NA07_Roizmannbacteria_52_60 | 0.004357 | 0.000658 | 0.0006 | 0.005614 |
| LAC_NA07_Truepera_radiovictrix_72_29 | 0.32921 | 0.212072 | 0.11242 | 0.653702 |
| LAC_NA08_Chloroflexi_58_8 | 0.441165 | 0.292891 | 0.302911 | 1.036966 |
| LAC_NA08_Chloroflexi_60_15 | 1.440842 | 0.427863 | 0.28559 | 2.154295 |
| LAC_NA08_Chloroflexi_67_31 | 0.315572 | 0.199258 | 0.411202 | 0.926032 |
| LAC_NA08_Flavobacteriales_41_5 | 0.030048 | 0.256915 | 0.131187 | 0.418151 |
| LAC_NA08_Gammaproteobacteria_68_22 | 0.030248 | 0.025794 | 0.01755 | 0.073592 |
| LAC_NA08_Ignavibacterium_album_33_266 | 14.10886 | 17.76094 | 23.01864 | 54.88844 |
| LAC_NA09_Acidobacteria_70_95 | 0.121235 | 0.043512 | 0.248251 | 0.412998 |
| LAC_NA09_Gammaproteobacteria_70_24 | 0.000345 | 0.001912 | 0.037637 | 0.039895 |
| LAC_NA10_Actinobacteria_74_12 | 0.069063 | 0.106258 | 0.30697 | 0.482291 |
| LAC_NA10_Bacteria_62_9 | 0.001946 | 0.002261 | 0.069944 | 0.074151 |
| LAC_NA10_Chloroflexi_62_239 | 9.181714 | 12.35382 | 15.9766 | 37.51214 |
| LAC_NA10_Lysobacter_70_8 | 0.014105 | 0.071352 | 0.113695 | 0.199152 |
| LAC_NA10_Xanthomonadales_68_7 | 0.01465 | 0.128817 | 0.112009 | 0.255475 |
| LAC_NA11_Bacteria_34_14 | 0.001217 | 0.000799 | 0.071575 | 0.073591 |
| LAC_NA11_Bacteria_57_35 | 0.00012 | 0.000471 | 0.000397 | 0.000989 |
| LAC_NA11_Bacteroidales_40_6 | 0.012362 | 0.099346 | 0.05127 | 0.162977 |
| LAC_NA11_Betaproteobacteria_62_13 | 0.000246 | 0.000402 | 0.028601 | 0.029249 |
| LAC_NA11_Caldilinea_aerophila_60_6 | 0.052892 | 0.07633 | 0.306149 | 0.435372 |
| LAC_NA11_Ignavibacteriales_56_75 | 0.001156 | 0.004679 | 0.023713 | 0.029547 |
| LAC_NA11_Microgenomates_50_64 | 0.091262 | 0.614933 | 1.127105 | 1.8333 |
| NA01_Acidobacteria_59_12 | 0.059256 | 0.094173 | 0.145765 | 0.299194 |
| NA01_Bacteria_60_23 | 0.009344 | 0.027154 | 0.084536 | 0.121033 |
| NA01_Caldilinea_aerophila_61_10 | 0.138865 | 0.055083 | 0.118356 | 0.312303 |
| NA01_Chloroflexi_65_16 | 0.853347 | 0.402605 | 0.240791 | 1.496744 |
| NA01_Fimbriimonas_ginsengisoli_61_16 | 0.046217 | 0.026844 | 0.056038 | 0.129099 |
| NA01_Rhizobiales_69_9 | 0.055615 | 0.200284 | 0.288574 | 0.544473 |
| NA01_Roizmanbacteria_38_28 | 0.006454 | 0.005005 | 0.036326 | 0.047784 |
| NA02_Anaerolinea_thermophila_56_16 | 0.00723 | 0.014861 | 0.177577 | 0.199668 |
| NA02_Rhizobiales_66_27 | 0.742663 | 0.448988 | 1.010378 | 2.202028 |
| NA03_Alphaproteobacteria_58_599 | 0.021953 | 0.018951 | 0.01113 | 0.052034 |
| NA03_Alphaproteobacteria_61_24 | 7.94E-05 | 0.001738 | 0.003421 | 0.005238 |
| NA03_Anaerolineales_41_23 | 0.218985 | 0.266058 | 0.657177 | 1.14222 |
| NA03_Bacteria_65_16 | 0.000105 | 0.000119 | 0.000285 | 0.000508 |
| NA03_BJP_IG2069_Ignavibacteriae_38_11_31_13 | 0.002173 | 0.00495 | 0.00077 | 0.007893 |
| NA03_Leptonema_illini_45_7 | 0.029639 | 0.039308 | 0.026414 | 0.095362 |
| NA03_RBG_16_Chloroflexi_57_11_curated_55_21 | 0.062375 | 0.083842 | 0.456644 | 0.602861 |
| NA04_Bacteria_50_51 | 0.004698 | 0.01877 | 0.037412 | 0.06088 |
| NA04_Bacteria_58_19 | 0.182447 | 0.288477 | 0.201094 | 0.672019 |
| NA04_Bacteria_65_10 | 0.03248 | 0.068478 | 0.124895 | 0.225853 |
| NA04_Bacteria_69_30 | 0.054346 | 0.044211 | 0.104293 | 0.202849 |
| NA04_Bacteroidetes_30_9 | 0.005584 | 0.071644 | 0.084641 | 0.161868 |
| NA04_BJP_IG2103_Bacteroidetes_37_22_46_30 | 0.000465 | 0.038325 | 0.013532 | 0.052323 |
| NA04_Burkholderiales_70_137 | 2.925124 | 2.992179 | 4.087406 | 10.00471 |
| NA04_Candidatus_Saccharibacteria_41_123 | 0.00104 | 0.000679 | 0.023445 | 0.025164 |
| NA04_Myxococcales_72_13 | 0.000137 | 0.002139 | 0.003983 | 0.006259 |
| NA04_Rhodocyclales_67_27 | 0.268306 | 0.581367 | 1.155097 | 2.00477 |
| NA04_Sphingobacteriales_44_8 | 0.003063 | 0.027064 | 0.031978 | 0.062106 |
| NA04_Turneriella_parva_44_15 | 0.000869 | 0.004679 | 0.010115 | 0.015663 |

|  | Upper Limit | Lower Limit |
| --- | --- | --- |
| Day 37 | 0.935288 | 0.051942 |
| Day 140 | 0.660955 | 0.07046 |
| Day 232 | 0.775536 | 0.050206 |

SI Table 9: MAG RPKM Confidence Intervals

Data Availability:

Raw metagenome sequences, metadata, and assembled genomes can be found on the ggkbase public repository: <https://ggkbase.berkeley.edu/project_groups/anamox_bioreactor_alvarez_cohen>

The 16S rRNA gene datasets analyzed during the current study are available on the Figshare repository: <https://figshare.com/account/home#/projects/169460>. All additional data is either available in the article, supplementary files, or available upon request.
